# SLC46A1 deficiency-mediated folate restriction suppresses colorectal cancer progression through epigenetic-transcriptional reprogramming

**DOI:** 10.1038/s41419-026-08423-8

**Published:** 2026-01-31

**Authors:** Yelu Zhou, Yanxing Liu, Yi Liu, Chang Che, Yihan Zhao, Jianing Yu, Xinhang Li, Ang Li, Shuyi Chen, Haojia Wang, Mingzhen Zhou, Dan Liu, Wenfang He, Zhuo Wang, Hua Han, Xin Wang, Yuanyuan Lu, Kaichun Wu, Xiaodi Zhao

**Affiliations:** 1 State Key Laboratory of Holistic Integrative Management of Gastrointestinal Cancers, National Clinical Research Center for Digestive Diseases, Xijing Hospital of Digestive Diseases, Xijing Hospital, Fourth Military Medical University, Xi’an, 710032 Shaanxi China; 2 Department of Nephrology, PLA 960th Hospital, Jinan, 250031 China; 3 Department of Biochemistry and Molecular Biology, Fourth Military Medical University, Xi’an, 710032 Shaanxi China; 4 Department of Gastroenterology, Tangdu Hospital, Fourth Military Medical University, Xi’an, 710038 Shaanxi China

**Keywords:** Cancer epidemiology, Oncogenes

## Abstract

The association between folate metabolism abnormalities and the development of colorectal cancer (CRC) remains controversial. Here, we report that the folate exerts a tumor-suppressive role in CRC; however, the manifestation of this effect is restricted by the expression level of folate transporter SLC46A1 in CRC cells. Multi-cohort profiling revealed significant downregulation of SLC46A1 in CRC tissues compared to adjacent normal tissues, where low expression independently predicted poor overall survival. Functional studies demonstrated that SLC46A1-mediated folate uptake suppressed tumor proliferation, migration, and invasion both in vitro and in vivo. Mechanistically, SLC46A1 deficiency restricted intracellular folate availability and impaired cellular methylation potential, as evidenced by a reduced SAM/SAH ratio, leading to DNA hypomethylation at specific sites such as the FOS proto-oncogene promoter. This epigenetic reprogramming triggers transcriptional activation of key oncogenic effectors CCND1, BCL2, and PLAU involved in CRC progression. Clinically, we found a significant inverse correlation between SLC46A1 expression and folate levels in tumor interstitial fluids of CRC, suggesting impaired folate uptake in low SLC46A1 tumors. Multi-color immunofluorescence across two cohorts further demonstrated conserved inverse associations between SLC46A1 and FOS expression in primary tumors and metastatic lesions. This study elucidates the molecular mechanism by which folate inhibits CRC progression through the “SLC46A1-epigenetic-transcriptional regulation” axis, providing mechanistic insights into folate deficiency-driven CRC progression and biomarkers for precision CRC intervention.

**Figure:**
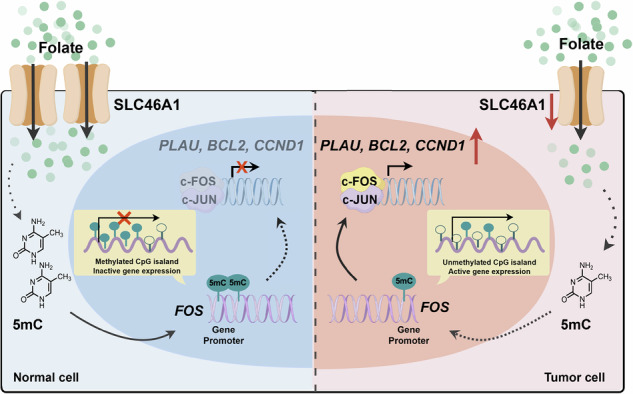
This study elucidates the tumor-suppressive role of the folate transporter SLC46A1 in CRC. In normal cells, SLC46A1 facilitates folate uptake, supporting one-carbon metabolism and maintaining genomic stability. In CRC, however, SLC46A1 downregulation induces intracellular folate deficiency, triggering locus-specific DNA hypomethylation at the FOS promoter, which activates oncogenic transcription of key downstream effectors (CCND1, BCL2, PLAU), driving tumor progression. The graphical abstract illustrates the differential impact of SLC46A1 on folate metabolism and gene expression in normal versus tumor cells, highlighting its potential as a therapeutic target in CRC.

This study elucidates the tumor-suppressive role of the folate transporter SLC46A1 in CRC. In normal cells, SLC46A1 facilitates folate uptake, supporting one-carbon metabolism and maintaining genomic stability. In CRC, however, SLC46A1 downregulation induces intracellular folate deficiency, triggering locus-specific DNA hypomethylation at the FOS promoter, which activates oncogenic transcription of key downstream effectors (CCND1, BCL2, PLAU), driving tumor progression. The graphical abstract illustrates the differential impact of SLC46A1 on folate metabolism and gene expression in normal versus tumor cells, highlighting its potential as a therapeutic target in CRC.

## Introduction

Colorectal cancer (CRC) remains a leading cause of cancer-related mortality globally [1, 2] with metabolic reprogramming emerging as a hallmark of its progression. Malignant cells meet their varying demands for metabolites by modulating the transport of these substances [3, 4] and the activity of key enzymes within metabolic cycles [5, 6]. This metabolic manipulation facilitates malignant progression, including tumor proliferation, invasion, metastasis, drug resistance, and angiogenesis [7–9]. Despite these effects, the precise molecular mechanisms that initiate and sustain these metabolic shifts and their role in CRC development are not yet completely understood.

Folate-mediated one-carbon metabolism is crucial for cellular processes, including nucleotide synthesis for cell division and the maintenance of methylation balance, which is essential for gene expression and genomic stability [10, 11]. Paradoxically, epidemiological studies present conflicting evidence: while folate deficiency increases CRC risk, excessive folate supplementation may paradoxically accelerate tumorigenesis in established lesions. Some malignant cells rely on excess folate metabolism to fulfill their biosynthetic needs, which has prompted the development of inhibitors like methotrexate and pralatrexate that have shown efficacy in treating hematological cancers [12, 13]. However, elevated folate metabolism is not observed in all tumor types. Evidence suggests that folate levels are significantly lower in intestinal adenomas and CRC tissues than in non-cancerous adjacent tissues, indicating that folate deficiency may be characteristic of CRC [14, 15]. This deficiency can disrupt DNA synthesis and repair, leading to DNA damage and genomic instability, which can promote tumorigenesis [16]. Low folate levels have been correlated with reduced DNA methylation, potentially accelerating tumor progression [17]. This dual role suggests context-dependent regulatory mechanisms within the folate metabolic network, yet the precise molecular determinants governing this dichotomy remain elusive.

Mammalian cells are incapable of de novo folate synthesis and rely on the uptake of this essential nutrient from extracellular sources via specific transporters or receptors, including folate receptors (FRs) and solute carrier transporters (SLC46A1 and SLC19A1) [18]. Our previous research demonstrated that tumor cells overexpress SLC6A6 to uptake taurine, leading to T-cell death and dysfunction, which, in turn, promotes tumor progression [19]. This finding has heightened our interest in the potential pivotal roles of SLC46A1 and SLC19A1 in the pathogenesis of CRC. Notably, of all folate transporters, SLC46A1 has been identified as the primary transporter for intestinal folate absorption and operates most efficiently at acidic pH values of 5.0–5 [20]. Given that the tumor microenvironment often becomes acidic due to factors such as hypoxia, SLC46A1 may play a more critical role in this context. However, the functions and mechanisms of SLC46A1 in CRC remain to be fully elucidated.

Existing studies on folate-CRC interactions have predominantly focused on extracellular folate bioavailability or catalytic enzymes (e.g., DHFR, MTHFR) [21–23], overlooking the pivotal role of transporters in shaping intracellular folate pools. Critically, intracellular folate levels directly dictate the availability of methyl donors for DNA methylation—an epigenetic mechanism frequently hijacked in cancer to silence tumor suppressors or activate oncogenes [24]. However, no study has systematically linked SLC46A1-mediated folate transport to locus-specific epigenetic reprogramming and transcriptional control in CRC.

Here, we demonstrate that folate itself exerts a tumor-suppressive effect in CRC, but this effect is critically dependent on the expression level of its transporter, SLC46A1. We show that SLC46A1 downregulation restricts intracellular folate availability and impairs cellular methylation capacity, triggering DNA hypomethylation specifically at the FOS proto-oncogene promoter. This epigenetic switch drives transcriptional activation of key oncogenic effectors, including CCND1, BCL2, and PLAU, thereby promoting CRC progression. Crucially, clinical validation across primary tumors and metastatic lesions reveals conserved inverse correlations between SLC46A1 expression and intratumoral folate levels or FOS activation. Our findings suggest that SLC46A1 holds promise as both a potential prognostic biomarker and a mechanistic linchpin in the “folate-epigenetic-transcriptional” axis driving CRC progression, laying a foundation for its future clinical application.

## Materials and methods

### Human specimens and ethics

Samples in CRC cohort 1 (*n* = 87), including CRC tissues and their paired adjacent non-tumor tissues, were obtained in the form of a tissue microarray from Outdo Biotech Co., Ltd. (Shanghai, China), a professional human biospecimen bank. Samples in CRC cohort 2 (*n* = 29), containing primary and metastatic CRC tissues, and samples in CRC cohort 3 (*n* = 40) were obtained at Xijing Hospital (Xi’an, China). All patients were pathologically diagnosed with CRC. The folate levels of 34 CRC samples in cohort 3 were measured in interstitial fluid based on sample availability and quality. The pathological and clinical information of all three cohorts are shown in Supplementary Table S1.

This study was conducted in accordance with the Declaration of Helsinki. The collection and research use of samples in cohort 1 were approved by the Independent Ethics Committee of Shanghai Outdo Biotech Company (Approval No. YB M-05-02). The collection and use of samples in cohorts 2 and 3 were approved by the Ethics Committee of Xijing Hospital (No. KY20222147-F-1). Written informed consent was obtained from all participants. This study does not contain identifiable images of participants; therefore, separate consent for image publication was not required.

Publicly available datasets analyzed in this study were obtained from The Cancer Genome Atlas (TCGA, https://cancergenome.nih.gov/) via the UCSC Xena browser (https://xena.ucsc.edu/) and from the Gene Expression Omnibus (GEO) database under accession number GSE156451.

### Mice

All animal experiments were conducted in accordance with the National Institutes of Health Guide for the Care and Use of Laboratory Animals, and were approved by the Fourth Military Medical University Experimental Animal Ethics Committee (KY20230590). BALB/c nude mice (female, aged 5–7 weeks) were purchased from Beijing Vital River Laboratory Animal Technology and housed in the Experimental Animal Center of the Fourth Military Medical University in accordance with institutional animal care guidelines. Mice were maintained under specific pathogen-free conditions with a 12-h light/dark cycle and provided with food and water ad libitum. Group sizes are specified in the figure legends. Mice were randomly assigned to each group using a random number generator.

### Cell culture

The human CRC cell lines DiFi, HCT8, SW480, HCT15, HCT116, DLD-1, and HT29 were obtained from the American Type Culture Collection; KM12C was obtained from the Coffey laboratory (Vanderbilt University). Cells were cultured in Dulbecco’s Modified Eagle Medium supplemented with 10% fetal bovine serum (FBS, Gibco), 1% Penicillin-Streptomycin (Gibco), and 1% L-glutamine in a 37 °C, 5% CO_2_ incubator. For experiments involving folate treatment, cells were cultured in folate-free medium (Pricella) as the control condition and in folate-free medium supplemented with a concentration of 100 nM folate (Sigma) as the experimental group. Cells were treated with 5 µM decitabine (MedChemExpress) for demethylation experiments.

### Transient transfection and lentiviral infection

A vector that expresses the complete open reading frame of SLC46A1 and its corresponding empty control were synthesized by GeneChem for in vitro transfection. siRNAs against SLC46A1, FOS, PLAU and their negative controls were purchased from GenePharma. Lentiviral shRNA targeting SLC46A1 was designed and packaged by GeneChem. Stable cell lines were generated by infecting the indicated cells with lentiviruses and selecting with puromycin. Infection efficiency was validated by qRT-PCR and western blotting. Plasmids or oligonucleotides were transfected using GP-transfect-Mate (GenePharma) reagents. All sequence information is shown in Supplementary Table S2.

### Cell proliferation assay

Cells were plated in a 96-well plate at a density of 3 × 10³ cells per well and subjected to the specified treatments. Cell proliferation was assessed at 24, 48, 72, 96, and 120 h after plating. At each time point, CCK-8 reagent was added to the wells (1:10 ratio), followed by a 2.5-h incubation. The absorbance at 450 nm was measured using a Varioskan Flash microplate reader (Thermo Fisher Scientific).

### Transwell migration and invasion assays

Cells after the indicated treatments were seeded in the upper chambers of Transwell inserts (Millipore) with (invasion assay) or without (migration assay) Matrigel at 1.5 × 10⁵ cells per well, with 20% FBS medium added to the bottom chamber. After incubating for 16–48 h at 37 °C, the cells that migrated or invaded to the lower surface were fixed with 4% paraformaldehyde, stained with 0.1% crystal violet, and imaged. Cells from five random non-overlapping fields per well were counted.

### In vivo tumor growth and metastasis assays

For subcutaneous tumor models, 5 × 10⁶ luciferase-labeled DiFi or HCT8 cells with stable SLC46A1 knockdown (using shSLC46A1#2) or control cells suspended in 100 µL of phosphate-buffered saline (PBS) were injected subcutaneously into the flanks of nude mice. Tumor volume was measured with calipers every 3 days starting from day 10, calculated using the formula: Volume = (L × W²)/2, where L is the length and W is the width. Body weight and general health status were recorded every 2–3 days. When the tumor volume reached ~1500 mm³, the mice were euthanized, and the tumor volume and weight were measured. The resected tumors were fixed in 4% paraformaldehyde, sectioned at 7 μm thickness, and subjected to hematoxylin and eosin (H&E) staining and immunohistochemical (IHC) staining for Ki-67 to assess tumor proliferation.

To investigate the effect of folate on tumor growth, nude mice were fed a folate-deficient diet prior to subcutaneous injection of DiFi cells. The folate dosing regimen was based on the common human therapeutic dose (10 mg/70 kg adult). The mouse systemic equivalent dose was calculated as 1.3 mg/kg via body surface area normalization and then adjusted for local delivery using a factor of 0.6 to establish a baseline dose of 0.78 mg/kg. Based on this, test doses of 0.8, 1.6, and 3.2 mg/kg (~1×, 2×, and 4× the baseline dose, respectively) were selected to examine the dose-response effect. Starting from day 10 after tumor inoculation, intratumoral injections of folate (dissolved in 20 µL of 5% DMSO per mouse) or vehicle control were administered every two days until the end of the experiment.

Metastasis model establishment and evaluation: HCT8 cells stably downregulating SLC46A1 and labeled with luciferase (5 × 10⁶ cells in 100 µL PBS) were injected into nude mice via the tail vein. Eight weeks later, bioluminescence intensity was analyzed using an IVIS Spectrum imaging system (PerkinElmer). The mice were then euthanized, and lung tissues were collected and fixed in 4% paraformaldehyde. Tissue sections were prepared and stained with H&E for counting metastatic nodules.

### RNA isolation and qRT-PCR

Total RNA was extracted using the MolPure® Cell/Tissue Total RNA Kit (Yeasen, Shanghai, China). RNA concentration and purity were determined by measuring the A260/A280 ratio. Complementary DNA (cDNA) was synthesized from 1 µg of total RNA using the 5× PrimeScript RT Master Mix (Takara). qPCR was performed using a SYBR Premix Ex Taq Kit (Takara) and a CFX96 QPCR Detection System (Bio-Rad). GAPDH was used as the internal control. Relative gene expression was calculated using the 2^–ΔΔCT method. Detailed primer information is shown in Supplementary Table S3.

### Protein isolation and western blot analysis

Proteins were extracted from the specified cells employing RIPA buffer (Beyotime) containing protease and phosphatase inhibitor (Roche). Then, they were separated by sodium dodecyl sulfate-polyacrylamide gel electrophoresis and blotted onto nitrocellulose membranes (Millipore). The membranes were blocked with 5% nonfat milk and then incubated with the primary antibodies and subsequently with secondary antibodies. Protein bands were detected using the enhanced Chemiluminescence Kit (Pierce) and visualized using the Molecular Imager ChemiDoc XRS+ Imaging System (Bio-Rad). Detailed primary antibody information is shown in Supplementary Table S4.

### In situ multi-color immunofluorescence staining and scoring

The tissue microarrays were heated at 60–65 °C for ~3 h, deparaffinized, and rehydrated. Endogenous peroxidase activity was blocked. Then, multiple cycles of antigen repairing, primary antibody incubation, polymer HRP-conjugated secondary antibody incubation, and tyramine signal amplification (TSA) dye staining were performed according to the instructions [25]. The staining intensity and the positive staining percentage was used to quantify indicated protein expression. Staining intensity was rated on a scale of 0–3, where 0 represented negative staining, 1 denoted weak staining, 2 indicated moderate staining, and 3 for strong staining. The proportion of cells displaying positive staining was determined semi-quantitatively. The staining intensity was multiplied by the positive cell percentage to calculate the histological score (H-score). The PerkinElmer Vectra 3 Imaging system was used to acquire the images. Scoring was performed by two independent pathologists who were blinded to the sample groups.

### Immunohistochemical (IHC) staining

IHC was performed on tissue microarrays and xenograft tumor sections. Section underwent deparaffinization and heat-induced antigen retrieval. Subsequently, the slides were treated with 3% H₂O₂ to block endogenous peroxidase activity. Afterward, they were incubated overnight at 4 °C with the primary antibody followed by incubation with HRP-conjugated secondary antibodies. Finally, visualization was achieved using 3,3-diaminobenzidine (DAB). Nuclei were counterstained with hematoxylin. The staining intensity was evaluated in the same way as for the in situ multi-color immunofluorescence staining intensity.

### RNA-seq and data analysis

Total RNA was extracted from the specified cells using TRIzol reagent (Invitrogen) and submitted to Shanghai Majorbio Bio-Pharm Technology Co., Ltd. for transcriptome sequencing. Library preparation and sequencing were performed on an Illumina NovaSeq 6000 platform following a standardized RNA quality control process. Differentially expressed genes were identified based on the criteria of |fold change|> 1.5 and adjusted *p*-value < 0.05.

### Tumor interstitial fluid (TIF) collection

Fresh tumor tissues were washed with PBS, minced into small pieces, and placed on a 70-µm cell strainer (Corning) seated in a 15-mL centrifuge tube. The samples were centrifuged at 400 × *g* for 15 min at 4 °C. The collected TIF was then filtered through a 0.22-µm syringe filter (Millipore) and stored at −80 °C until analysis.

### Folate measurement

Folate levels were measured using platform-appropriate assays. The folate concentration in TIF samples from CRC patients was measured using a competitive Enzyme-Linked Immunosorbent Assay (ELISA) kit (Cloud-Clone Corp., Katy, USA), according to the manufacturer’s guidelines. Absorbance was measured at 450 nm using a Thermo Scientific Varioskan Flash reader. Intracellular folate levels in a subset of cell lines under SLC46A1 knockdown and control conditions were additionally analyzed using a more specific High-Performance Liquid Chromatography with Tandem Mass Spectrometry (HPLC-MS/MS) method, as described below.

### Determination of folate by HPLC-MS/MS

Chromatographic separation was achieved using a Waters HSS T3 column (3.0 mm × 100 mm, 1.8 µm) on an Agilent 1290 Infinity II HPLC system. The mobile phase consisted of 0.1% formic acid in water (A) and acetonitrile (B). The gradient elution program was: 0–0.2 min, 3% B; 0.2-3 min, 3%–70% B; 0.3–3.0 min, 70%–100% B; 3.0–4.8 min, 100% B; 4.8–5.4 min, 3% B. The flow rate was 0.4 mL/min, and the injection volume was 2 µL. Mass spectrometric detection was performed on an AB SCIEX 3200 QTRAP mass spectrometer with an electrospray ionization (ESI) source operated in positive ion mode. The source parameters were as follows: ion spray voltage, 5500 V; temperature, 550 °C; nebulizer gas (Gas 1), 55 psi; auxiliary gas (Gas 2), 60 psi; curtain gas, 35 psi. Folic acid was quantified using multiple reaction monitoring (MRM) of the transitions *m/z* 440.0 → 175.1 (declustering potential, −60 V; collision energy, −50 V) and *m/z* 440.0 → 132.0 (declustering potential, −60 V; collision energy, −67 V), with the former used for quantification. The method was validated for linearity, precision, and accuracy over the expected concentration range in biological samples.

### Determination of SAM and SAH by HPLC-MS/MS

S-adenosylmethionine (SAM) and S-adenosylhomocysteine (SAH) were analyzed using a Kinetex HILIC column (2.1 mm × 100 mm, 2.6 µm) on an Agilent 1290 Infinity II system. The mobile phase was 20 mM ammonium formate in water, pH 3.5 (A), and 20 mM ammonium formate in 90% acetonitrile, pH 3.5 (B). The gradient was: 0–2.5 min, 100% B; 2.5–3.5 min, 95% B; 3.5–6.0 min, 60% B; 6.0–5 min, 45% B; 6.5–8.0 min, 45% B; 8.0–1 min, 100% B; 8.1–10.0 min, 100% B. Detection was carried out on the AB SCIEX 3200 QTRAP system. The ESI source parameters were identical to those described in Section “Determination of folate by HPLC-MS/MS”. SAM and SAH were monitored using MRM transitions: SAM, *m/z* 399.1 → 250.2 (declustering potential, 80 V; collision energy, 23 V); SAH, *m/z* 385.1 → 134.2 (declustering potential, 75 V; collision energy, 28 V). The method was validated for linearity (R² ≥ 0.99), repeatability, and accuracy.

### DNA isolation and global DNA methylation measurement

Genomic DNA was extracted from the cell lines using a DNeasy Blood & Tissue Kit (Qiagen). The global DNA methylation level was assessed by measuring 5-methylcytosine (5-mC) content using a commercial ELISA kit (Epigentek) according to the manufacturer’s instructions. The 5-mC level was normalized to the total DNA input.

### Bisulfite pyrosequencing (BSP) & next-generation sequencing (NGS)

Genomic DNA was isolated using a DNeasy Blood & Tissue Kit (Qiagen), and the samples were then submitted to Shanghai Sangon Biotech for analysis of methylation levels at CpG sites in promoter regions by bisulfite pyrosequencing and next-generation sequencing on an Illumina MiSeq platform.

### Statistical analysis

Statistical analyses were performed using GraphPad Prism (v9.0), SPSS (v26.0), and R (v4.0.0), with data presented as mean ± SD. Normality and variance homogeneity were verified where appropriate. For all analyses, effect sizes and 95% confidence intervals (CIs) are reported, and statistical significance was defined as a two-sided *P* < 0.05. Group comparisons were analyzed by Student’s *t* tests (reporting Cohen’s d), one-way, or two-way ANOVA with post-hoc tests (reporting η²). Categorical data were assessed by Chi-squared or Fisher’s exact test (reporting odds ratios, OR). Correlations were determined using Pearson’s coefficient (r). Survival analysis was performed using the Kaplan–Meier method and log-rank test, with hazard ratios (HR) and 95% confidence intervals (CI) derived from Cox proportional hazards models. A two-sided *p*-value of <0.05 was considered statistically significant. For RNA-seq data, the Benjamini–Hochberg procedure was used to control the false discovery rate (FDR).

## Results

### SLC46A1 is the major transporter for folate in colon and is downregulated in CRC tissues and correlated with poor prognosis

**Fig. 1:**
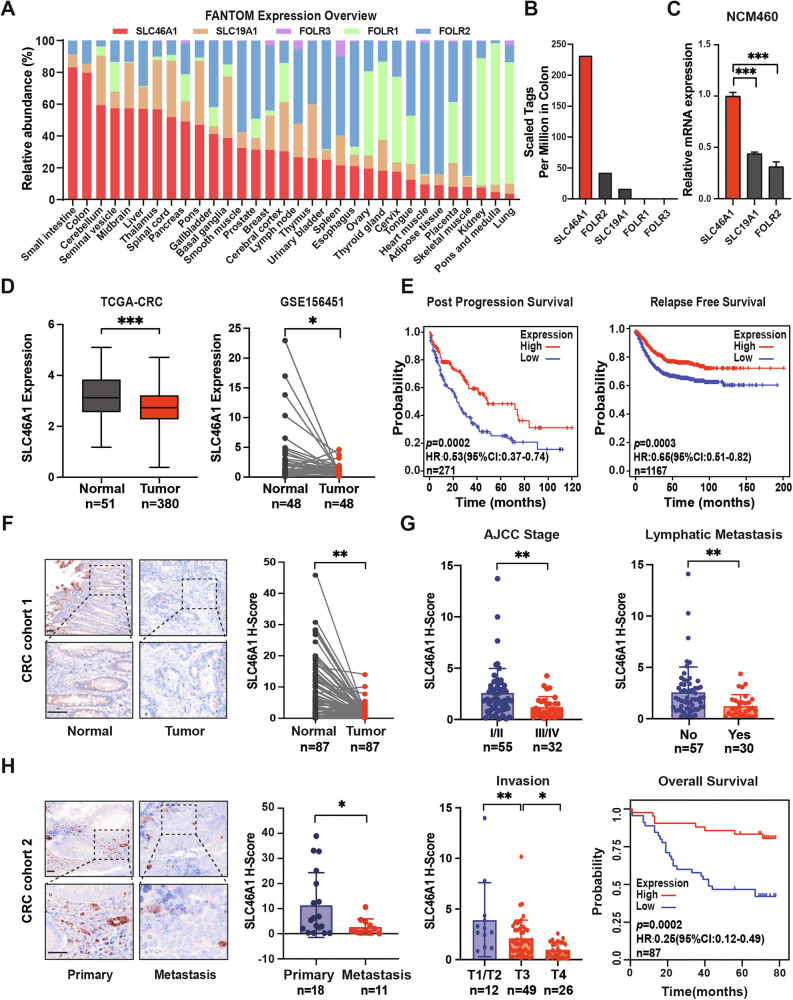
SLC46A1 is downregulated in human CRC tissues and correlated with poor prognosis. **A** The relative abundance of folate transporters in diverse human tissue types determined by the Function Annotation of the Mammalian Genome (FANTOM) project. **B** The scaled tags of folate transporters in colon determined by the FANTOM project. **C** The relative mRNA expression of SLC46A1, SLC19A1, FOLR2 in NCM460 cells. **D** Expression levels of SLC46A1 in CRC tissues compared with unpaired (left) or paired (right) adjacent non-tumor tissues, as analyzed in the TCGA-CRC and GEO databases. **E** Kaplan–Meier survival curves for patients with low and high SLC46A1 expression in the TCGA-CRC database. **F** Representative IHC staining and quantification of SLC46A1 protein in 87 paired CRC and adjacent tissues. **G** Correlation between SLC46A1 expression and prognostic factors, along with Kaplan–Meier survival curves for patients with low and high SLC46A1 expression in CRC cohort 1 (*n* = 87). **H** Representative IHC staining and quantification of SLC46A1 in primary (*n* = 18) and metastatic (*n* = 11) CRC lesions. Bar plot data are presented as the mean ± SD. Statistical significance was assessed using unpaired t-test (**D** left), paired *t* test (**D** right, **F**, **G** upper and **H**), one-way ANOVA (**C**, **G** lower) and log rank test (**E**, **G** lower). HR and 95% CI from Cox regression. *p < 0.05, **p < 0.01, ***p < 0.001. Scale bars: upper, 20 µm; lower, 50 µm. SLC46A1 Solute carrier family 46 member 1, CRC colorectal cancer, TCGA the cancer genome atlas, GEO the gene expression omnibus, IHC immunohistochemistry, HR hazard ratio, CI confidence interval, SD standard deviation.

To determine the expression levels of different folate transporters in the human colon, we first analyzed tissue-specific expression data from the FANTOM project. This analysis revealed that SLC46A1 was the predominant folate transporter in the colon, with a high relative expression level of 79.7%. In contrast, the other widely studied folate transporters, FOLR1 and SLC19A1, showed minimal expression (0.07% and 5.68%, respectively) (Fig. 1A). Among them, SLC46A1, FOLR2 and SLC19A1 were the top three folate transporters in the colon (Fig. 1B). To verify these foundings, we examined the expression of SLC46A1, FOLR2 and SLC19A1 in human normal colon cell lines NCM460 and FHC by RT-qPCR. Consistent with the bioinformatics data, SLC46A1 expression was markedly higher than that of FOLR2 and SLC19A1, confirming its role as the major folate transporter in colon cells (Fig. 1C and Supplementary Fig. S2A).

Based on these results, we focused on SLC46A1 and investigated its expression in CRC. Analysis of the TCGA and GSE156451 datasets showed that SLC46A1 was significantly downregulated in CRC tissues compared to non-tumor tissues (Fig. 1D). Kaplan–Meier survival analysis revealed that lower SLC46A1 expression was associated with shorter post-progression survival (PPS, *p* = 0.0002) and relapse-free survival (RFS, *p* = 0.0003) in CRC patients (Fig. 1E). In contrast to normal colon cell lines, the expression of SLC46A1 was decreased in most of the CRC cell lines (Supplementary Fig. S2B). This was further supported by IHC staining in the CRC tissue microarray (CRC cohort 1), which demonstrated decreased SLC46A1 expression in CRC tumor tissues compared to adjacent normal mucosa (Fig. 1F). In normal epithelium, SLC46A1 staining was frequently observed at or near the plasma membrane, whereas in tumor cells, the overall signal intensity was markedly reduced, with any residual staining presenting a diffuse, cytoplasmic pattern. We further observed a negative correlation between SLC46A1 expression and aggressive CRC phenotypes, including pathological grade, lymphatic metastasis, and tumor stage, and higher SLC46A1 expression was associated with improved overall survival, as determined in the CRC cohort 1 analysis (Fig. 1G). Furthermore, in another CRC tissue microarray (CRC cohort 2), the expression of SLC46A1 was significantly reduced in metastatic lesions compared to their matched primary tumors (Fig. 1H). Additionally, analysis of the TCGA database revealed that the expression of SLC46A1 was consistently lower in various types of cancer compared to adjacent non-cancerous tissues and was highly correlated with patient prognosis (Supplementary Fig. S1A, B). Collectively, these findings suggest that SLC46A1 might exhibit tumor-suppressing properties in CRC development and progression.

### SLC46A1 inhibits the proliferation, migration, and invasion of colorectal cancer cells

**Fig. 2:**
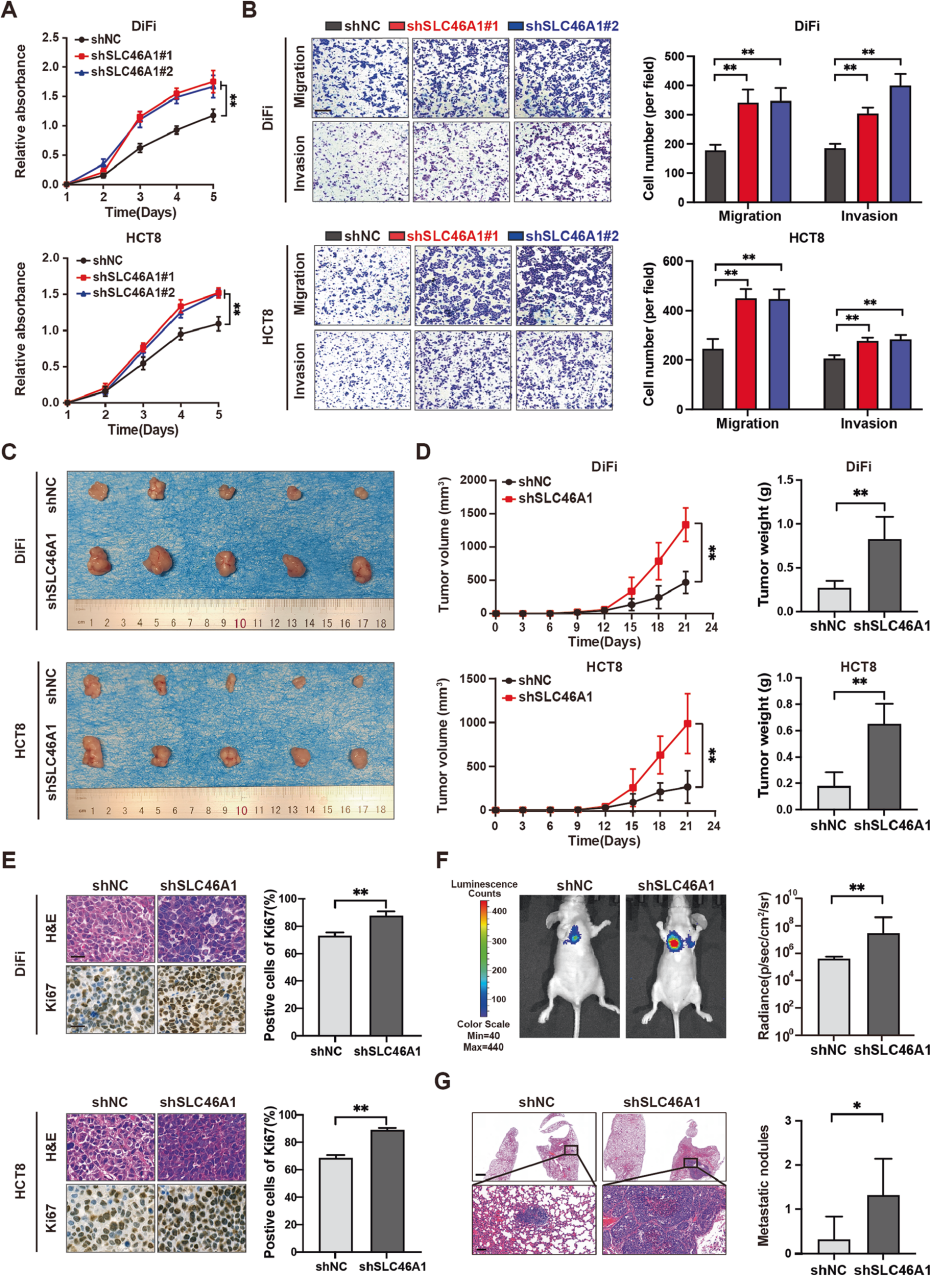
SLC46A1 inhibits the proliferation, migration, and invasion of colorectal cancer cells. **A** Growth curves of DiFi and HCT8 cells with SLC46A1 inhibition. **B** Representative images of migration and invasion assays and quantitative analysis of DiFi and HCT8 cells under SLC46A1 inhibition; scale bar 200 μm. **C** Macroscopic views showing the xenografts in the indicated subgroups. **D** Tumor volume progression and tumor weights in xenograft models in the indicated groups (*n* = 5). **E** Representative H&E and Ki-67 immunohistochemical staining images of subcutaneous xenograft tumors, with corresponding quantitative analysis of Ki-67-positive cells (*n* = 5. scale bar, 50 μm). **F** Bioluminescence images and radiance level measurements of lung metastasis at 8 weeks after HCT8 cell injection into the tail vein of nude mice. **G** H&E staining images of the lungs of nude mice and quantitative graph of metastatic nodules (scale bar, upper 1000 μm, lower 100 μm). Bar plot data are expressed as the mean ± SD. Significant differences were assessed by repeated measures two-way ANOVA (**A**, **D** left), one-way ANOVA (**B**), or Student’s *t* test (right panels of **D**–**G**). *p < 0.05, **p < 0.01. H&E Hematoxylin and eosin, SD standard deviation.

Based on the observed downregulation of SLC46A1 in CRC and its association with aggressive phenotypes and poor prognosis, we hypothesized that SLC46A1 functions as a tumor suppressor in CRC. To test this hypothesis, we assessed its functional impact on key oncogenic behaviors: proliferation, migration, and invasion. Based on endogenous expression levels, DiFi and HCT8 cells (high SLC46A1) were selected for knockdown experiments, while KM12C and SW480 cells (low SLC46A1) were chosen for overexpression experiments (Supplementary Fig. S2B). Lentiviral and plasmid transfection was used to modulate SLC46A1 expression, which was verified by qRT-PCR and western blotting (Supplementary Fig. S2C–E). Functional assays revealed that SLC46A1 knockdown significantly enhanced the proliferation of DiFi and HCT8 cells (CCK-8 assay; Fig. 2A). Furthermore, transwell assays demonstrated that SLC46A1 depletion markedly increased both migration and invasion in these cells (Fig. 2B). Conversely, SLC46A1 overexpression suppressed invasion in KM12C and SW480 cells (Supplementary Fig. S2F).

Consistent with the in vitro findings, in vivo studies showed that subcutaneous injection of SLC46A1-knockdown (shSLC46A1) DiFi and HCT8 cells into nude mice resulted in significantly increased tumor volume and weight compared to control (shNC) cells (Fig. 2C, D). IHC staining of these tumors confirmed enhanced proliferation, as evidenced by increased Ki-67 positivity in the shSLC46A1 group (Fig. 2E). Bioluminescence imaging following tail vein injection of HCT8-shSLC46A1 or HCT8-shNC cells revealed significantly higher lung metastatic potential in the knockdown group (Fig. 2F). This was corroborated by H&E staining, which showed more numerous and larger metastatic lung lesions in mice receiving shSLC46A1 cells (Fig. 2G). Importantly, this functional reliance on SLC46A1 was non-redundant, as its knockdown did not elicit compensatory upregulation of other folate transporters (SLC19A1, FOLR1, FOLR2), as confirmed by RT‑qPCR in vitro and further supported by analysis of the GSE87211 cohort in vivo (Supplementary Fig. S2G, H). Collectively, these results indicate that loss of SLC46A1 enhances proliferative and metastatic potential in CRC cells both in vitro and in vivo.

### Folate inhibits CRC progression in a SLC46A1-dependent manner

**Fig. 3:**
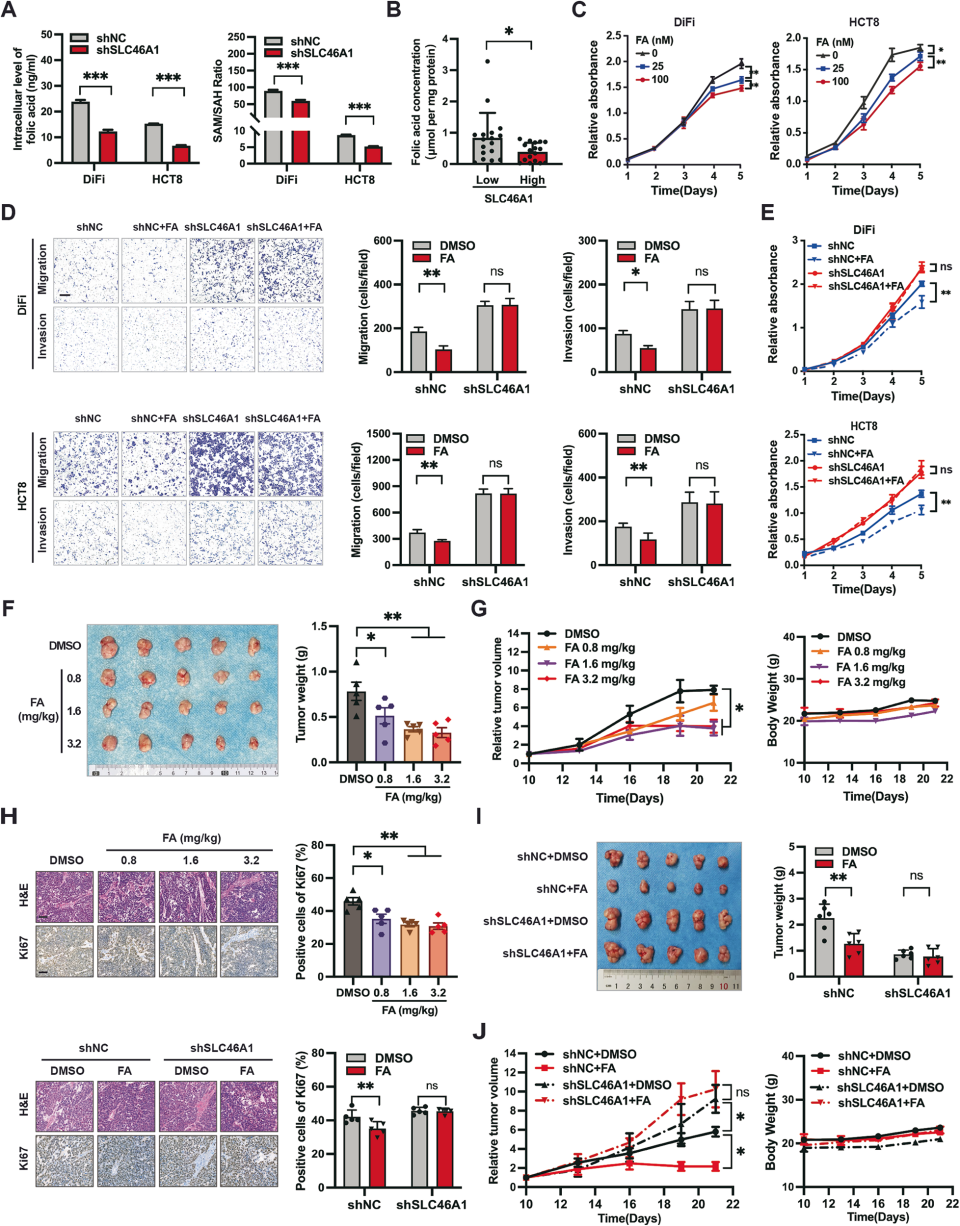
SLC46A1 inhibits CRC progression by transporting folate in vitro and in vivo. **A** Intracellular folate levels and the SAM/SAH ratio in control and SLC46A1-knockdown DiFi and HCT8 cells, quantified by HPLC-MS/MS. **B** Folate concentration in tumor interstitial fluid (TIF) from Cohort 3 (*n* = 34), determined by ELISA, shows an inverse correlation with SLC46A1 expression. **C** Growth curves of DiFi and HCT8 cells treated with 0, 25, and 100 nM folate. **D** Representative images of migration and invasion assays and quantitative analysis of DiFi and HCT8 cells under SLC46A1 inhibition treated with PBS or 100 nM folate, scale bar 200 μm. **E** Growth curves of DiFi and HCT8 cells under SLC46A1 restriction treated with PBS or 100 nM folate. **F**, **G** Macroscopic views, tumor weights, tumor volume progression, and body weight changes of DiFi xenograft models treated with different concentrations of folate (*n* = 5 per group). **H** Representative H&E and Ki67 staining images and quantitative analysis of Ki67 positivity rate of subcutaneous tumors (*n* = 5 per group; scale bar, 100 μm). **I**, **J** Macroscopic views, tumor weights, tumor volume progression, and body weight changes of DiFi xenograft models in indicated groups treated with 1.6 mg/kg folate. Bar plot data are expressed as the mean ± SD. Significant differences were assessed by repeated measures two-way ANOVA (**C**, **E**, left panels of **G** and **H**), one-way ANOVA (**A**, **D**, right panels of **G**, **J**-**L**), or Student’s *t* tests (**B**). *p < 0.05, **p < 0.01, ***p < 0.001. FA Folate acid, H&E Hematoxylin and eosin, SD standard deviation.

Given the role of SLC46A1 in mediating cellular folate uptake, we examined whether its tumor-suppressive function involves folate transport. Knockdown of SLC46A1 using shRNAs in DiFi and HCT8 cells resulted in reduced intracellular folate levels relative to control shRNA-treated cells. Consistent with impaired folate availability, we observed a significant decrease in the SAM/SAH ratio—a key metric of methylation capacity—following SLC46A1 silencing (Fig. 3A), indicating a decline in methyl donor supply. We further analyzed tumor interstitial fluid (TIF) from a cohort of CRC patients and found that folate levels in TIF were inversely correlated with SLC46A1 expression: lower in tumors with high SLC46A1 and higher in those with low expression (Fig. 3B), supporting its role as a principal folate transporter in CRC.

We next assessed folate’s effects on the malignant behavior of CRC cells. Based on the physiological folate concentration in human serum (13.5–45.3 nM) [26, 27], we chose 0, 25, and 100 nM to treat DiFi and HCT8 cells that were cultured in folate-free media. Folate supplementation suppressed proliferation, migration, and invasion in both cell lines. Crucially, these anti-tumor effects were abolished upon SLC46A1 knockdown (Fig. 3C–E), confirming the necessity of SLC46A1 for folate-mediated suppression of malignancy. We further validated these findings in vivo using a localized intervention approach in folate-deficient mice bearing DiFi xenografts. Intratumoral folate injection significantly inhibited tumor growth compared to saline controls (Fig. 3F, G), and IHC revealed reduced Ki-67⁺ proliferation (Fig. 3H). This local administration regimen did not induce systemic toxicity, as evidenced by stable body weights and normal histology in major organs (Fig. 3G, Supplementary Fig. S3A). In contrast, xenografts derived from SLC46A1-knockdown DiFi cells were unresponsive to folate, showing neither growth suppression nor reduced Ki-67 expression (Fig. 3H–J). Together, these results demonstrate that the tumor-suppressive effects of folate in colorectal cancer are strictly dependent on SLC46A1-mediated cellular uptake.

### SLC46A1 inhibits FOS expression in a DNA methylation-dependent manner

**Fig. 4:**
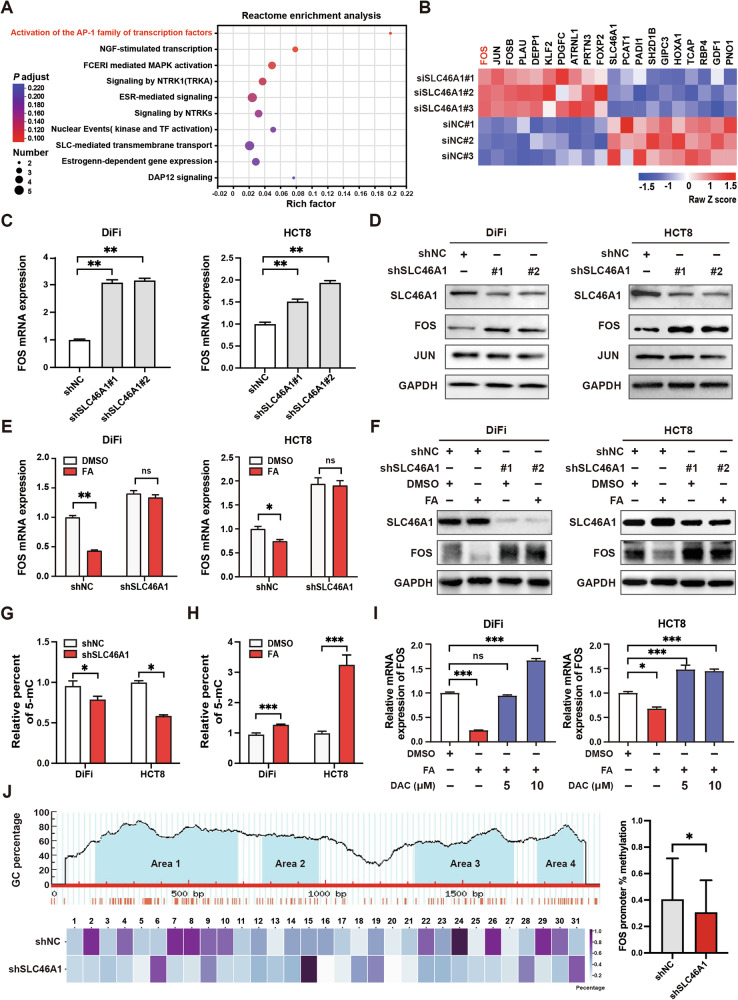
SLC46A1 inhibits FOS expression in folate-dependent and DNA methylation-dependent manners. **A** Reactome enrichment analysis of changed pathways in SLC46A1-downregulated HCT8 cells. **B** Heatmap of the altered genes in HCT8 cells inhibiting SLC46A1 compared with negative controls. **C**, **D** FOS mRNA and protein levels after shSLC46A1 transfection in DiFi and HCT8 cells. **E**, **F** FOS mRNA and protein levels in SLC46A1-inhibited cells treated with folate or PBS. **G** Global 5-mC levels in SLC46A1-downregulated DiFi and HCT8 cells. **H** Global 5-mC levels in DiFi and HCT8 cells treated with folate or PBS. **I** FOS mRNA levels in DiFi and HCT8 cells treated with folate and DAC or DMSO. **J** CpG island prediction in the FOS promoter region using MethPrimer; changes in FOS promoter DNA methylation (left) and quantitative analysis of CpG methylation within Area 1 of the FOS promoter after SLC46A1 knockdown (right). Bar plot data are expressed as the mean ± SD. Significant differences were assessed by one-way ANOVA (**C**, **I**) or Student’s *t* tests (**E**, **G**, **H**, **J** right). *p < 0.05, **p < 0.01, ***p < 0.001. Decitabine (DAC), Enzyme-linked immunosorbent assay (ELISA), 5-methylcytosine (5-mC), next-generation sequencing bisulfite pyrosequencing (NGS-BSP).

To investigate how SLC46A1 regulates CRC progression, we performed RNA sequencing in HCT8 cells after SLC46A1 knockdown. This identified 729 differentially expressed genes (DEGs; |fold change|> 1.5, adjusted *p*-value < 0.05), indicating broad transcriptional reprogramming. Reactome pathway analysis of these DEGs revealed enrichment in several oncogenic pathways, with “activation of AP-1 family transcription factors” being the most significantly affected (Fig. 4A). Given the established role of AP-1 in oncogenesis and the pronounced enrichment of this pathway, we prioritized it for further investigation. Indeed, RNA-seq data confirmed the marked upregulation of multiple AP-1 family members, including FOS, JUN, and FOSB (Fig. 4B), an effect corroborated by RT-qPCR in both DiFi and HCT8 cells (Supplementary Fig. S3B). Among these, FOS showed the most consistent and marked upregulation at both mRNA and protein levels, as confirmed by qRT-PCR (Fig. 4C) and western blot (Fig. 4D). Therefore, by integrating the global transcriptomic profile with mechanistic validation, we identified FOS as a key downstream effector of SLC46A1 signaling.

Given that SLC46A1 is the major folate transporter in the colon and folate serves as a cofactor in one-carbon metabolism—essential for DNA methylation—we proposed that SLC46A1 regulates FOS expression via folate-mediated epigenetic mechanisms. Folate treatment (100 nM) significantly downregulated FOS expression in both DiFi and HCT8 cells compared to PBS controls, an effect that was reversed by SLC46A1 knockdown (Fig. 4E, F). Global DNA methylation was reduced following SLC46A1 depletion (Fig. 4G) and increased with folate supplementation (Fig. 4H). Decitabine (DAC), a DNA methyltransferase inhibitor, abolished folate-induced FOS downregulation (Fig. 4I), confirming the involvement of DNA methylation. To identify specific regulatory regions, we analyzed the FOS promoter using MethPrimer and located four CpG islands. Bisulfite sequencing revealed hypomethylation specifically in Area 1 of the FOS promoter in SLC46A1-knockdown DiFi cells. Quantitative analysis of all CpG sites within Area 1 showed a significant decrease in average methylation after SLC46A1 knockdown (Fig. 4J). In contrast, promoter methylation of JUN and FOSB was largely unaffected (Supplementary Fig. S3C, D). Together, these results indicate that SLC46A1-mediated folate uptake represses FOS expression by sustaining hypermethylation at a specific promoter region, unveiling a novel epigenetic pathway in CRC progression.

### SLC46A1 inhibits proliferation, migration, and invasion by suppressing FOS-mediated transcriptional regulation

**Fig. 5:**
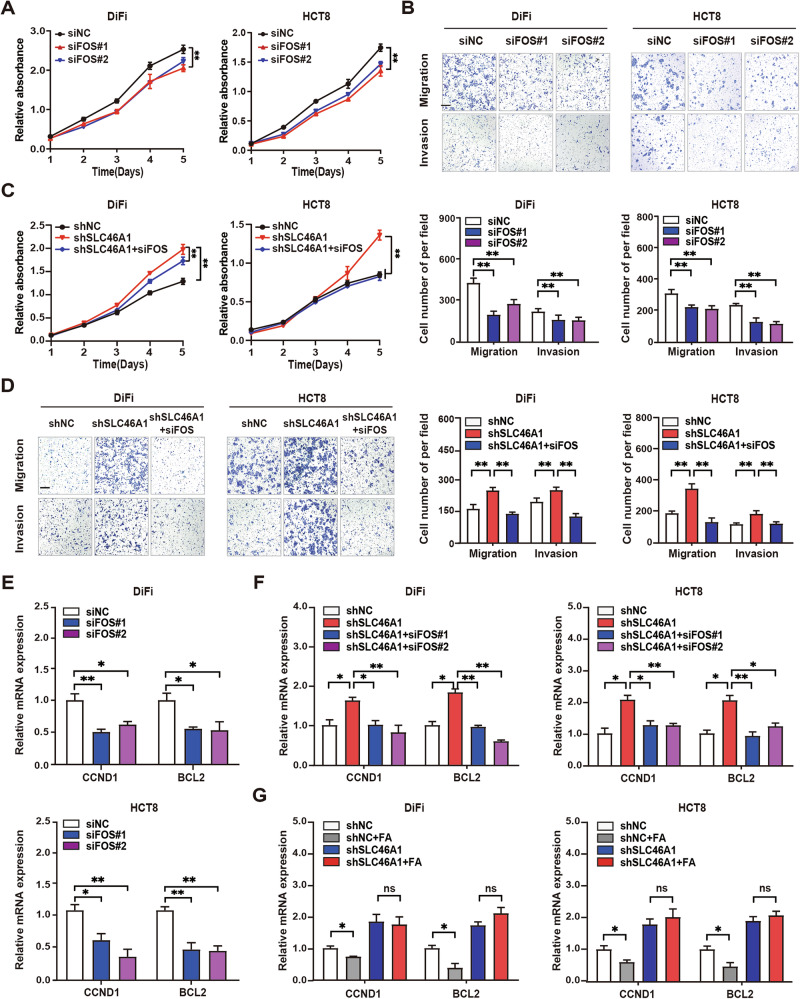
SLC46A1 inhibits proliferation by suppressing FOS-mediated CCND1 and BCL2 transcription. **A** Growth curves of DiFi and HCT8 cells with suppressed FOS expression. **B** Representative images of migration and invasion assays and quantitative analysis of DiFi and HCT8 cells with inhibited FOS expression. Scale bars, 200 μm. **C** Growth curves of SLC46A1-suppressed DiFi and HCT8 cells with or without FOS inhibition. **D** Representative images of migration and invasion assays and quantitative analysis of SLC46A1-suppressed DiFi and HCT8 cells with or without FOS inhibition. Scale bars, 200 μm. **E** CCND1 and BCL2 mRNA levels with or without FOS repression. **F** CCND1 and BCL2 mRNA in SLC46A1-inhibited cells with or without FOS repression. **G** CCND1 and BCL2 mRNA in SLC46A1-inhibited cells treated with folate or PBS. Bar plot data are expressed as the mean ± SD. Significant differences were assessed by repeated measures two-way ANOVA (**A**, **C**), one-way ANOVA (**B**, **D**-**F**) and Student’s *t* test (**G**). *p < 0.05, **p < 0.01. FA Folate acid, SD standard deviation.

To determine whether FOS mediates the tumor-promoting effects of SLC46A1, we first evaluated the functional consequences of FOS knockdown in CRC cells. Transient silencing of FOS with siRNAs (siFOS) in HCT8 and DiFi cells markedly reduced proliferation, migration, and invasion compared to controls (Fig. 5A, B; Supplementary Fig. S3E), indicating that FOS is essential for maintaining oncogenic phenotypes. Rescue experiments further demonstrated that FOS depletion completely abolished the enhanced proliferation and migration induced by SLC46A1 knockdown (Fig. 5C, D), establishing FOS as a critical downstream effector of SLC46A1 in CRC progression.

Given FOS’s function as a transcription factor, we systematically identified its downstream targets mediating cancer progression. Combining bioinformatic analysis of AP-1 binding motifs with literature curation, we focused on two proliferation-associated genes: CCND1 (cell cycle regulator) and BCL2 (anti-apoptotic factor). FOS knockdown markedly reduced both CCND1 and BCL2 mRNA levels (Fig. 5E). Notably, the transcriptional upregulation of these genes following SLC46A1 silencing was fully reversed upon concomitant FOS depletion (Fig. 5F), confirming their regulation through the SLC46A1-FOS axis. To assess the physiological relevance of folate-mediated regulation, we performed supplementation experiments in folate-deficient medium. Restoration of folate concentrations (100 nM) suppressed *CCND1* and *BCL2* expression in control cells, but this suppression was significantly attenuated in SLC46A1-deficient cells (Fig. 5G), further linking folate metabolism to FOS-dependent transcriptional control.

**Fig. 6:**
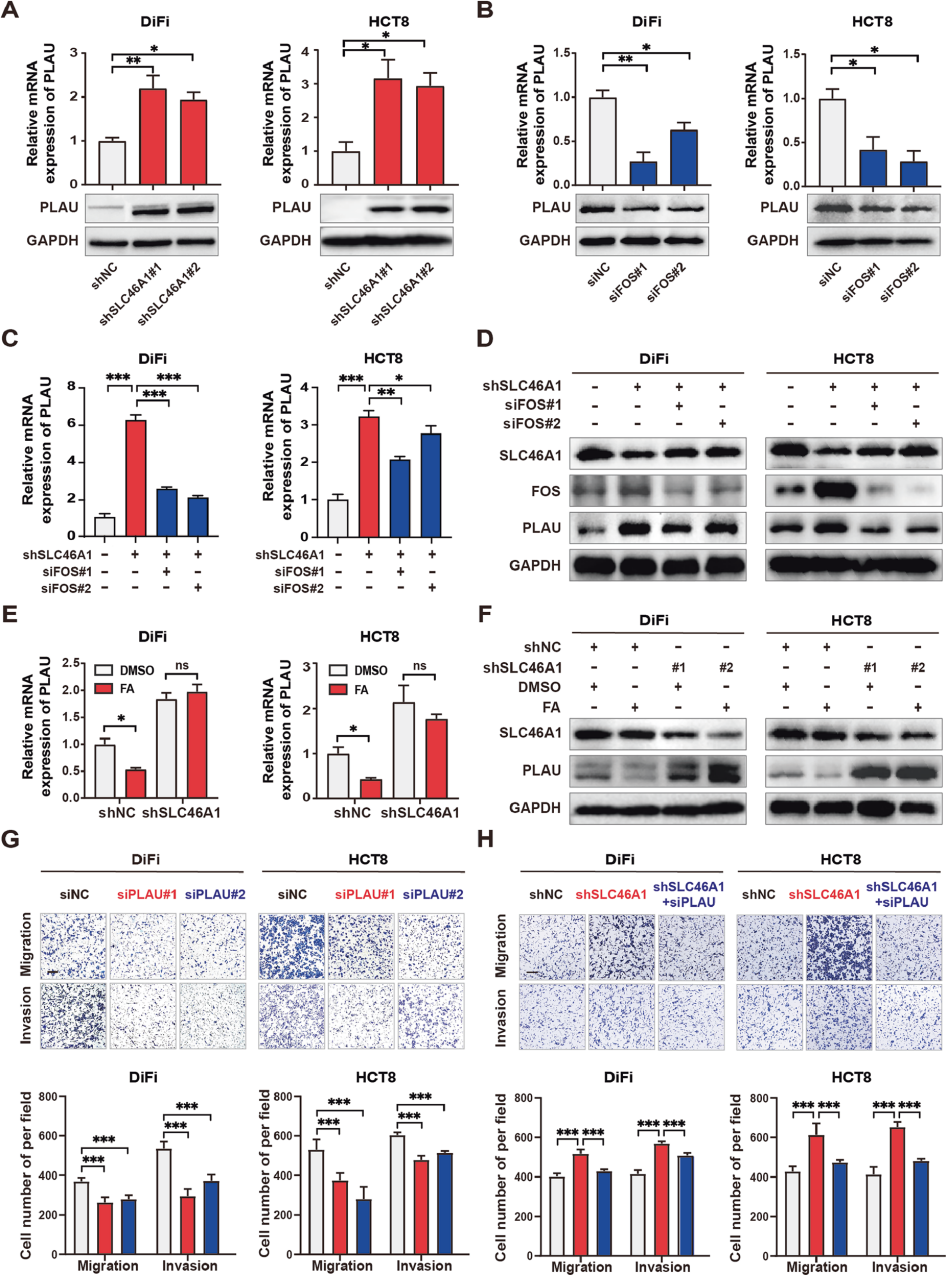
SLC46A1 inhibits metastasis by suppressing FOS-mediated PLAU transcription. PLAU mRNA and protein expression levels in DiFi and HCT8 cells following SLC46A1 knockdown (**A**) or FOS knockdown (**B**). **C**, **D** PLAU mRNA and protein expression levels in SLC46A1-knockdown DiFi and HCT8 cells, with or without concomitant FOS knockdown. **E**, **F** PLAU mRNA and protein expression levels in SLC46A1-knockdown DiFi and HCT8 cells treated with folate (100 nM) or vehicle control (PBS). **G** Representative images and quantitative analysis of migration and invasion assays in DiFi and HCT8 cells with or without PLAU knockdown. **H** Representative images and quantitative analysis of migration and invasion assays in SLC46A1-knockdown DiFi and HCT8 cells with or without concomitant PLAU knockdown. Scale bars, 200 μm. Bar plot data are expressed as the mean ± SD. Significant differences were assessed by one-way ANOVA. *p < 0.05, **p < 0.01, ***p < 0.001.

We also identified PLAU (encoding urokinase plasminogen activator, a key protease for extracellular matrix remodeling) as an AP-1 target gene upregulated upon SLC46A1 suppression (Fig. 4B). SLC46A1 knockdown increased both PLAU mRNA and protein levels (Fig. 6A), while FOS silencing mitigated this upregulation (Fig. 6B). Dual knockdown of SLC46A1 and FOS restored PLAU expression to baseline (Fig. 6C, D). Consistent with folate’s regulatory role, physiological folate supplementation suppressed PLAU in control cells but not in SLC46A1-depleted cells (Fig. 6E, F). Functionally, PLAU knockdown (Supplementary Fig. S3F) impaired cell migration and invasion (Fig. 6G), and co-silencing SLC46A1 and PLAU reversed the pro-metastatic phenotype caused by SLC46A1 loss (Fig. 6H), suggesting that PLAU is a key downstream mediator of SLC46A1-mediated metastasis suppression. Together, these findings indicate that SLC46A1 acts as a tumor suppressor by promoting folate uptake, which in turn represses FOS-dependent transcriptional programs involving CCND1, BCL2, and PLAU, thereby inhibiting proliferation, migration, and invasion in CRC cells.

### SLC46A1 and FOS exhibit clinically relevant inverse correlation during colorectal cancer progression

**Fig. 7:**
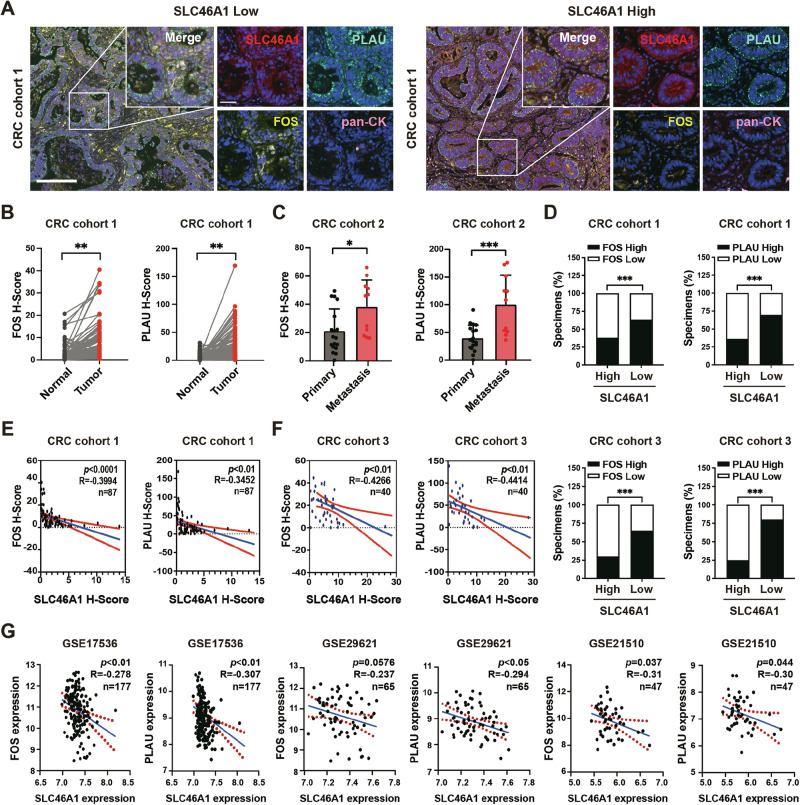
SLC46A1 is negatively correlated with FOS and PLAU in the CRC cohort. **A** Representative SLC46A1, FOS, PLAU and pan-CK immunofluorescence staining in CRC tissues from cohort 1. Scale bar, 200 μm (lower) and 50 μm (upper). Expression levels of FOS and PLAU mRNA in CRC cohort 1 (**B**) and cohort 2 (**C**). **D** Percentages of FOS-positive and PLAU-positive cases in the high and low SLC46A1 expression groups of cohort 1 and cohort 3. Correlation between SLC46A1 expression and the expression of FOS and PLAU in cohort 1 (**E**) and cohort 3 (**F**). **G** Correlation between SLC46A1 expression and the expression of FOS and PLAU in GSE17536, GSE29621 and GSE21510. Bar plot data are expressed as the mean ± SD. Significant differences were assessed by Student’s *t* test (**B**, **C**), Pearson’s χ2 test (**D**), and Pearson’s correlation analysis (**E**–**G**). **p < 0.01, ***p < 0.001.

To bridge mechanistic discovery with clinical relevance, we first assessed the protein levels of SLC46A1, FOS, and PLAU using multiplex immunohistochemistry. In CRC Cohort 1 (87 paired primary tumors and adjacent tissues), both FOS and PLAU were significantly enriched in tumor tissues compared with normal epithelia (Fig. 7A, B). Moreover, metastatic lesions (Cohort 2: 18 primaries vs. 11 metastatic samples) showed further elevated expression of FOS and PLAU (Fig. 7C), suggesting pathway activation during metastatic progression. Notably, SLC46A1 expression was inversely correlated with both FOS and PLAU in Cohort 1 and Cohort 3 (Fig. 7D–F). This consistent inverse relationship was independently validated in GEO datasets (Fig. 7G). Importantly, multivariate Cox regression analysis of the GSE39582 cohort (*n* = 585), adjusted for age, gender, chemotherapy status, tumor location, and TNM stage, confirmed that high SLC46A1 expression serves as an independent prognostic factor associated with better overall survival (HR = 0.44, 95% CI: 0.22–91, *p* = 0.026; Table 1). Together, these clinical findings validate our experimental model and establish SLC46A1 downregulation as a clinically relevant event driving FOS/PLAU activation and tumor progression in CRC.

**Table 1:** Multivariate Cox regression analysis of overall survival in the public validation cohort (GSE39582, *n* = 585).

Variable	Category	HR (95% CI)	*p*-value
SLC46A1	High vs Low^a^	0.44 (0.22–0.91)	0.026
Age, years	≥60 vs <60	1.19 (0.81–1.74)	0.381
Sex	Male vs Female	1.30 (0.93–1.82)	0.123
Adjuvant Chemotherapy	Yes vs No	1.02 (0.68–1.54)	0.911
Tumor Location	Distal vs Proximal	1.29 (0.92–1.83)	0.143
Stage	III/IV vs I/II	2.23 (1.48–3.35)	<0.001

*HR* hazard ratio, *CI* confidence interval.

^a^The optimal cut-off value for SLC46A1 expression (High vs Low) was determined by maximally selected rank statistics.

## Discussion

Our study unveils a novel dual regulatory mechanism by which SLC46A1 exerts folate metabolism-dependent tumor-suppressive effects in colorectal carcinogenesis through an “epigenetic-transcriptional cascade”. This discovery reconciles longstanding controversies regarding the dichotomous role of folate in CRC pathogenesis [28–30], providing a mechanistic framework that integrates transporter biology with epigenetic regulation.

Previous studies have predominantly focused on the “biphasic modulation” of folate—characterized by its cancer-preventive effects in normal tissues through maintaining DNA stability and methylation equilibrium, versus its tumor-promoting role in neoplastic tissues by supplying nucleotide precursors to fuel proliferation [31–34]. However, this paradigm neglects the spatiotemporal orchestration of folate partitioning by transporters such as SLC46A1, which we identify as a master regulator converting systemic folate abundance into tumor-suppressive epigenetic signals through selective intracellular depletion. Crucially, under physiological folate concentrations (in contrast to supraphysiological levels used in prior models) [26, 27, 35], SLC46A1-mediated folate restriction triggers DNA hypomethylation at specific loci, particularly the FOS promoter. This localized epigenetic reprogramming suppresses oncogenic effectors (e.g., CCND1, BCL2, PLAU). Clinical validation in Cohort 3 reveals a striking inverse correlation between SLC46A1 expression and unutilized folate accumulation in tumor interstitial fluid. These findings not only provide a new perspective to understand the complex role of folate in tumor biology but also suggest potential applications for folate-based strategies in CRC treatment, especially in the context of personalized medicine.

Beyond resolving the folate paradox, our discovery of SLC46A1 as a rheostat controlling epigenetic-transcriptional coupling poses broader implications. While FOS/AP-1’s pro-tumorigenic role via CCND1/Bcl2/PLAU activation is established in multiple malignancies [36–38], the regulatory interface with folate metabolism remains novel. The spatially restricted folate depletion caused by SLC46A1 downregulation creates a unique metabolic vulnerability: while global hypomethylation may predispose to genomic instability, the targeted repression of oncogenic amplifiers like FOS tilts the balance toward tumor suppression. This DNA methylation-dependent regulation adds a new layer to the epigenetic control of FOS. Whereas previous studies showed that FOS drives tumor progression via epigenetic plasticity [39, 40], with PRMT1-mediated methylation stabilizing FOS in gastric cancer [41] and histone methylation modulating its expression [42]. Our work extends these paradigms to CRC, demonstrating SLC46A1 deficiency facilitates FOS upregulation through DNA hypomethylation.

These findings collectively position SLC46A1 as a putative therapeutic target and unveil several actionable avenues for future translation. First, from a nutritional and pharmacological perspective, our data suggest that the efficacy of folate-based interventions (e.g., dietary folate modulation or supplementation) is likely contingent upon tumor SLC46A1 expression levels. This insight could inform the development of biomarker-stratified nutritional guidelines or therapies, where patients with high tumor SLC46A1 might benefit more from folate supplementation, whereas those with low SLC46A1 would require alternative strategies. Second, a promising therapeutic approach could involve the direct targeting of the SLC46A1-epigenetic axis, either through pharmacological agents designed to upregulate or mimic SLC46A1 function, or more immediately, via rational combination therapies. For instance, combining DNA methyltransferase inhibitors (e.g., decitabine) with folate supplementation could be tested to determine if it synergistically reverses the aberrant hypomethylation and oncogene activation in SLC46A1-deficient tumors. Alternatively, co-targeting the vulnerable downstream effectors, such as using FOS/PLAU pathway antagonists, represents another viable combinatorial strategy. However, we acknowledge that translating these concepts faces considerable challenges. The feasibility of specifically modulating a folate transporter in vivo, ensuring targeted delivery to tumor tissue while avoiding systemic impacts on normal folate metabolism, remains a significant hurdle. Future studies employing inducible overexpression models, patient-derived organoids, and testing the proposed combination regimens are essential to formally validate these therapeutic hypotheses and assess whether restoring the SLC46A1-mediated epigenetic program can elicit a robust anti-tumor effect.

In addition, this study has several limitations that should be considered. First, while we identified SLC46A1 as the principal folate transporter in colorectal tissues and established its role in driving DNA hypomethylation, the broader chromatin-level effects—such as changes in histone modifications or transcription factor binding at loci such as FOS—remain unclear. Future studies employing epigenomic assays will be essential to fully delineate the consequences of folate disruption. Second, the potential functional redundancy among folate transporters, including SLC19A1 and FOLR1–3, has not been fully explored, leaving open the possibility of compensatory mechanisms in certain contexts. Finally, although murine xenograft models were informative, they may not fully capture systemic folate metabolism or human-specific tumor–host interactions. Subsequent work should therefore focus on validating these findings in more physiologically relevant models or clinical cohorts.

By elucidating SLC46A1’s role in coupling folate metabolism to epigenetic-transcriptional regulation, this work advances our understanding of CRC biology. The identified “SLC46A1-folate-FOS” axis provides a dual-target strategy for precision interventions—modulating folate availability in SLC46A1-low tumors or directly targeting FOS/PLAU effectors. These insights pave the way for biomarker-driven clinical trials integrating folate status and SLC46A1 expression into therapeutic decision-making.

## Supplementary information

Supplementary files

Reproducibility checklist

Supplementary Figure 1

Supplementary Figure 2

Supplementary Figure 3

Full and uncropped western blots

## Data Availability

The data supporting the findings of this study are available within the article and its supplementary information files. Additional data are available from the corresponding authors upon reasonable request.
