# Supplementary material for: SLC46A1 deficiency-mediated folate restriction suppresses colorectal cancer progression through epigenetic-transcriptional reprogramming
Source: Cell Death Dis. 2026 Jan 31;17(1):189. doi: 10.1038/s41419-026-08423-8 (PMC12876983; doi:10.1038/s41419-026-08423-8)
Supplement: Supplementary file 1 — Supplementary files [file 41419_2026_8423_MOESM1_ESM.docx]

**Supplementary Files**

**Supplementary Figure Legends**

**Figure S1. Analysis of SLC46A1 expression and its correlation with patient prognosis in** **various cancer types.** (A) Box plots showing the relative transcript levels of SLC46A1 in normal and tumor tissues in four different cancer types: lung squamous cell carcinoma (LUSC), pancreatic adenocarcinoma (PAAD), pheochromocytoma and paraganglioma (PCPG), and kidney renal clear cell carcinoma (KIRC). (B) Kaplan–Meier survival curves for patients with high and low SLC46A1 expression levels in the respective cancer types.

Significant differences were assessed by Student’s *t*-tests (A) and log rank test (B). HR and 95% CI from Cox regression. Bar plot data are presented as the mean ± SEM, ****p* < 0.001.

**Figure S2. Analysis of SLC46A1 expression and functional impact in CRC cells.** (A) Relative mRNA expression of SLC46A1, SLC19A1 and FOLR2 in FHC cell. (B) Relative SLC46A1 mRNA expression level in CRC cell lines compared with the human normal colorectal epithelial cell lines (FHC and NCM460). (C, D) SLC46A1 mRNA and protein expression in DiFi and HCT8 cells post-shRNA transfection. (E) SLC46A1 protein expression in SW480 and KM12C cells post-vector transfection. (F) Representative images and quantitative analysis of invasion assays of SW480 and KM12C cells after SLC46A1 overexpression, scale bar 200 μm. (G) Expression of folate transporters in SLC46A1-low human colorectal tumors from the GSE87211 cohort (n = 203). (H) mRNA expression of alternative folate transporters following SLC46A1 knockdown in DiFi and HCT8 cells. Bar plot data are expressed as the mean ± SD. Significant differences were assessed by one-way ANOVA (A, B, C, E, H) or Student’s *t*-tests (F, G). ***p* < 0.01, ****p* < 0.001.

**Figure S3. CpG locations of FOS, JUN, and FOSB and DNA methylation changes in JUN and FOSB.** (A) H&E staining images of major organs from xenograft mice following local folate administration. (B) Relative mRNA levels of AP-1 transcription factor family members upon SLC46A1 knockdown. (C) Schematic of CpG sites relative to the FOS gene locus. (D) Schematic of CpG sites relative to the FOSB gene locus and heatmap showing FOSB DNA methylation changes in SLC46A1-knockdown HCT8 cells. (E) Schematic of CpG sites relative to the JUN gene locus and heatmap showing JUN DNA methylation changes in SLC46A1-knockdown HCT8 cells. (F) FOS mRNA and protein expression levels in DiFi and HCT8 cells following siRNA-mediated knockdown. (G) PLAU mRNA and protein expression levels in DiFi and HCT8 cells following siRNA-mediated knockdown. Bar plot data are expressed as the mean ± SD. Significant differences were assessed by two-way ANOVA (B), Student’s *t*-tests (C, D) or one-way ANOVA (E, F). **p* < 0.05, ***p* < 0.01.

**Supplementary Table 1. Clinicopathological characteristics of CRC cohort.**

**CRC cohort 1** (Note: Data in the blue background is deleted data, due to tissue loss during staining.)

| **Site** | **Label** | **Histological type** | **Pathological type** | **Grade** |
| --- | --- | --- | --- | --- |
| A01 | D15A1454-B30-C1 | Colorectal cancer | Adenocarcinoma | Ⅱ |
| A02 | D15A1454-B30-P1 | Adjacent tissue | Mucosa of the colon |  |
| A03 | D15A1455-B30-C1 | Colorectal cancer | Adenocarcinoma | Ⅲ |
| A04 | D15A1455-B30-P1 | Adjacent tissue | Mucosa of the colon |  |
| A05 | D15A1456-B30-C1 | Colorectal cancer | Adenocarcinoma | Ⅱ |
| A06 | D15A1456-B30-P1 | Adjacent tissue | Mucosa of the colon |  |
| A07 | D15A1458-B30-C1 | Colorectal cancer | Adenocarcinoma | Ⅱ |
| A08 | D15A1458-B30-P1 | Adjacent tissue | Mucosa of the colon |  |
| A09 | D15A1516-B30-C1 | Colorectal cancer | Adenocarcinoma | Ⅱ |
| A10 | D15A1516-B30-P1 | Adjacent tissue | Mucosa of the colon |  |
| A11 | D15A1461-B30-C1 | Colorectal cancer | Adenocarcinoma | Ⅲ |
| A12 | D15A1461-B30-P1 | Adjacent tissue | Mucosa of the colon |  |
| A13 | D15A1464-B30-C1 | Colorectal cancer | Adenocarcinoma | Ⅲ |
| A14 | D15A1464-B30-P1 | Adjacent tissue | Mucosa of the colon |  |
| A15 | D15A1462-B30-C1 | Colorectal cancer | Adenocarcinoma | Ⅳ |
| A16 | D15A1462-B30-P1 | Adjacent tissue | Mucosa of the colon |  |
| A17 | D15A1502-B30-C1 | Colorectal cancer | Adenocarcinoma | Ⅱ |
| A18 | D15A1502-B30-P1 | Adjacent tissue | Mucosa of the colon |  |
| B01 | D15A1503-B30-C1 | Colorectal cancer | Adenocarcinoma | Ⅱ |
| B02 | D15A1503-B30-P1 | Adjacent tissue | Mucosa of the colon |  |
| B03 | D15A1504-B30-C1 | Colorectal cancer | Adenocarcinoma | Ⅰ |
| B04 | D15A1504-B30-P1 | Adjacent tissue | Mucosa of the colon |  |
| B05 | D15A1505-B30-C1 | Colorectal cancer | Adenocarcinoma | Ⅱ |
| B06 | D15A1505-B30-P1 | Adjacent tissue | Mucosa of the colon |  |
| B07 | D15A1508-B30-C1 | Colorectal cancer | Adenocarcinoma | Ⅱ |
| B08 | D15A1508-B30-P1 | Adjacent tissue | Mucosa of the colon |  |
| B09 | D15A1510-B30-C1 | Colorectal cancer | Adenocarcinoma | Ⅱ |
| B10 | D15A1510-B30-P1 | Adjacent tissue | Mucosa of the colon |  |
| B11 | D15A1556-B30-C1 | Colorectal cancer | Adenocarcinoma | Ⅱ |
| B12 | D15A1556-B30-P1 | Adjacent tissue | Mucosa of the colon |  |
| B13 | D15A1557-B30-C1 | Colorectal cancer | Adenocarcinoma | Ⅱ |
| B14 | D15A1557-B30-P1 | Adjacent tissue | Mucosa of the colon |  |
| B15 | D15A1558-B30-C1 | Colorectal cancer | Adenocarcinoma | Ⅲ |
| B16 | D15A1558-B30-P1 | Adjacent tissue | Mucosa of the colon |  |
| B17 | D15A1559-B30-C1 | Colorectal cancer | Adenocarcinoma | Ⅱ |
| B18 | D15A1559-B30-P1 | Adjacent tissue | Mucosa of the colon |  |
| C01 | D15A1560-B30-C1 | Colorectal cancer | Adenocarcinoma | Ⅰ |
| C02 | D15A1560-B30-P1 | Adjacent tissue | Mucosa of the colon |  |
| C03 | D15A1561-B30-C1 | Colorectal cancer | Adenocarcinoma | Ⅲ |
| C04 | D15A1561-B30-P1 | Adjacent tissue | Mucosa of the colon |  |
| C05 | D15A1562-B30-C1 | Colorectal cancer | Adenocarcinoma | Ⅳ |
| C06 | D15A1562-B30-P1 | Adjacent tissue | Mucosa of the colon |  |
| C07 | D15A1563-B30-C1 | Colorectal cancer | Adenocarcinoma | Ⅱ |
| C08 | D15A1563-B30-P1 | Adjacent tissue | Mucosa of the colon |  |
| C09 | D15A1564-B30-C1 | Colorectal cancer | Adenocarcinoma | Ⅱ |
| C10 | D15A1564-B30-P1 | Adjacent tissue | Mucosa of the colon |  |
| C11 | D15A1565-B30-C1 | Colorectal cancer | Adenocarcinoma | Ⅰ |
| C12 | D15A1565-B30-P1 | Adjacent tissue | Mucosa of the colon |  |
| C13 | D15A1566-B30-C1 | Colorectal cancer | Adenocarcinoma | Ⅰ |
| C14 | D15A1566-B30-P1 | Adjacent tissue | Mucosa of the colon |  |
| C15 | D15A1567-B30-C1 | Colorectal cancer | Adenocarcinoma | Ⅲ |
| C16 | D15A1567-B30-P1 | Adjacent tissue | Mucosa of the colon |  |
| C17 | D15A1570-B30-C1 | Colorectal cancer | Adenocarcinoma | Ⅲ |
| C18 | D15A1570-B30-P1 | Adjacent tissue | Mucosa of the colon |  |
| D01 | D15A1571-B30-C1 | Colorectal cancer | Adenocarcinoma | Ⅱ |
| D02 | D15A1571-B30-P1 | Adjacent tissue | Mucosa of the colon |  |
| D03 | D15A1572-B30-P1 | Colorectal cancer | Adenocarcinoma | Ⅲ |
| D04 | D15A1572-B30-C1 | Adjacent tissue | Mucosa of the colon |  |
| D05 | D15A1573-B30-C1 | Colorectal cancer | Adenocarcinoma | Ⅱ |
| D06 | D15A1573-B30-P1 | Adjacent tissue | Mucosa of the colon |  |
| D07 | D15A1574-B30-C1 | Colorectal cancer | Adenocarcinoma | Ⅱ |
| D08 | D15A1574-B30-P1 | Adjacent tissue | Mucosa of the colon |  |
| D09 | D15A1576-B30-C1 | Colorectal cancer | Adenocarcinoma | Ⅰ |
| D10 | D15A1576-B30-P1 | Adjacent tissue | Mucosa of the colon |  |
| D11 | D15A1577-B30-C1 | Colorectal cancer | Adenocarcinoma | Ⅲ |
| D12 | D15A1577-B30-P1 | Adjacent tissue | Mucosa of the colon |  |
| D13 | D15A1579-B30-P1 | Colorectal cancer | Adenocarcinoma | Ⅱ |
| D14 | D15A1579-B30-C1 | Adjacent tissue | Mucosa of the colon |  |
| D15 | D15A1614-B30-C1 | Colorectal cancer | Adenocarcinoma | Ⅱ |
| D16 | D15A1614-B30-P1 | Adjacent tissue | Mucosa of the colon |  |
| D17 | D15A1628-B30-C1 | Colorectal cancer | Adenocarcinoma | Ⅲ |
| D18 | D15A1628-B30-P1 | Adjacent tissue | Mucosa of the colon |  |
| E01 | D15A1615-B30-C1 | Colorectal cancer | Adenocarcinoma | Ⅰ |
| E02 | D15A1615-B30-P1 | Adjacent tissue | Mucosa of the colon |  |
| E03 | D15A1616-B30-C1 | Colorectal cancer | Adenocarcinoma | Ⅱ |
| E04 | D15A1616-B30-P1 | Adjacent tissue | Mucosa of the colon |  |
| E05 | D15A1617-B30-C1 | Colorectal cancer | Adenocarcinoma | Ⅱ |
| E06 | D15A1617-B30-P1 | Adjacent tissue | Mucosa of the colon |  |
| E07 | D15A1619-B30-C1 | Colorectal cancer | Adenocarcinoma | Ⅱ |
| E08 | D15A1619-B30-P1 | Adjacent tissue | Mucosa of the colon |  |
| E09 | D15A1620-B30-C1 | Colorectal cancer | Adenocarcinoma | Ⅱ |
| E10 | D15A1620-B30-P1 | Adjacent tissue | Mucosa of the colon |  |
| E11 | D15A1622-B30-C1 | Colorectal cancer | Adenocarcinoma | Ⅱ |
| E12 | D15A1622-B30-P1 | Adjacent tissue | Mucosa of the colon |  |
| E13 | D15A1629-B30-C1 | Colorectal cancer | Adenocarcinoma | Ⅱ |
| E14 | D15A1629-B30-P1 | Adjacent tissue | Mucosa of the colon |  |
| E15 | D15A1624-B30-C1 | Colorectal cancer | Adenocarcinoma | Ⅲ |
| E16 | D15A1624-B30-P1 | Adjacent tissue | Mucosa of the colon |  |
| E17 | D15A1625-B30-C1 | Colorectal cancer | Adenocarcinoma | Ⅱ |
| E18 | D15A1625-B30-P1 | Adjacent tissue | Mucosa of the colon |  |
| F01 | D15A1626-B30-C1 | Colorectal cancer | Adenocarcinoma | Ⅲ |
| F02 | D15A1626-B30-P1 | Adjacent tissue | Mucosa of the colon |  |
| F03 | D15A1630-B30-C1 | Colorectal cancer | Adenocarcinoma | Ⅰ |
| F04 | D15A1630-B30-P1 | Adjacent tissue | Mucosa of the colon |  |
| F05 | D15A1663-B30-C1 | Colorectal cancer | Adenocarcinoma | Ⅱ |
| F06 | D15A1663-B30-P1 | Adjacent tissue | Mucosa of the colon |  |
| F07 | D15A1668-B30-C1 | Colorectal cancer | Adenocarcinoma | Ⅲ |
| F08 | D15A1668-B30-P1 | Adjacent tissue | Mucosa of the colon |  |
| F09 | D15A1669-B30-C1 | Colorectal cancer | Adenocarcinoma | Ⅱ |
| F10 | D15A1669-B30-P1 | Adjacent tissue | Mucosa of the colon |  |
| F11 | D15A1732-B30-C1 | Colorectal cancer | Adenocarcinoma | Ⅱ |
| F12 | D15A1732-B30-P1 | Adjacent tissue | Mucosa of the colon |  |
| F13 | D15A1733-B30-C1 | Colorectal cancer | Adenocarcinoma | Ⅲ |
| F14 | D15A1733-B30-P1 | Adjacent tissue | Mucosa of the colon |  |
| F15 | D15A1735-B30-C1 | Colorectal cancer | Adenocarcinoma | Ⅱ |
| F16 | D15A1735-B30-P1 | Adjacent tissue | Mucosa of the colon |  |
| F17 | D15A1740-B30-C1 | Colorectal cancer | Adenocarcinoma | Ⅲ |
| F18 | D15A1740-B30-P1 | Adjacent tissue | Mucosa of the colon |  |
| G01 | D15A1741-B30-C1 | Colorectal cancer | Adenocarcinoma | Ⅲ |
| G02 | D15A1741-B30-P1 | Adjacent tissue | Mucosa of the colon |  |
| G03 | D15A1742-B30-C1 | Colorectal cancer | Adenocarcinoma | Ⅰ |
| G04 | D15A1742-B30-P1 | Adjacent tissue | Mucosa of the colon |  |
| G05 | D15A1745-B30-C1 | Colorectal cancer | Adenocarcinoma | Ⅱ |
| G06 | D15A1745-B30-P1 | Adjacent tissue | Mucosa of the colon |  |
| G07 | D15A1743-B30-C1 | Colorectal cancer | Adenocarcinoma | Ⅲ |
| G08 | D15A1743-B30-P1 | Adjacent tissue | Mucosa of the colon |  |
| G09 | D15A1744-B30-C1 | Colorectal cancer | Adenocarcinoma | Ⅳ |
| G10 | D15A1744-B30-P1 | Adjacent tissue | Mucosa of the colon |  |
| G11 | D15A1756-B30-C1 | Colorectal cancer | Adenocarcinoma | Ⅲ |
| G12 | D15A1756-B30-P1 | Adjacent tissue | Mucosa of the colon |  |
| G13 | D15A1758-B30-C1 | Colorectal cancer | Adenocarcinoma | Ⅱ |
| G14 | D15A1758-B30-P1 | Adjacent tissue | Mucosa of the colon |  |
| G15 | D15A1765-B30-C1 | Colorectal cancer | Adenocarcinoma | Ⅱ |
| G16 | D15A1765-B30-P1 | Adjacent tissue | Mucosa of the colon |  |
| G17 | D15A1767-B30-C1 | Colorectal cancer | Adenocarcinoma | Ⅱ |
| G18 | D15A1767-B30-P1 | Adjacent tissue | Mucosa of the colon |  |
| H01 | D15A1762-B30-C1 | Colorectal cancer | Adenocarcinoma | Ⅱ |
| H02 | D15A1762-B30-P1 | Adjacent tissue | Mucosa of the colon |  |
| H03 | D15A1764-B30-C1 | Colorectal cancer | Adenocarcinoma | Ⅲ |
| H04 | D15A1764-B30-P1 | Adjacent tissue | Mucosa of the colon |  |
| H05 | D15A1990-B30-C1 | Colorectal cancer | Adenocarcinoma | Ⅱ |
| H06 | D15A1990-B30-P1 | Adjacent tissue | Mucosa of the colon |  |
| H07 | D15A1811-B30-C1 | Colorectal cancer | Adenocarcinoma | Ⅱ |
| H08 | D15A1811-B30-P1 | Adjacent tissue | Mucosa of the colon |  |
| H09 | D15A1813-B30-C1 | Colorectal cancer | Adenocarcinoma | Ⅲ |
| H10 | D15A1813-B30-P1 | Adjacent tissue | Mucosa of the colon |  |
| H11 | D15A1814-B30-C1 | Colorectal cancer | Adenocarcinoma | Ⅰ |
| H12 | D15A1814-B30-P1 | Adjacent tissue | Mucosa of the colon |  |
| H13 | D15A1991-B30-C1 | Colorectal cancer | Adenocarcinoma | Ⅱ |
| H14 | D15A1991-B30-P1 | Adjacent tissue | Mucosa of the colon |  |
| H15 | D15A1815-B30-C1 | Colorectal cancer | Adenocarcinoma | Ⅱ |
| H16 | D15A1815-B30-P1 | Adjacent tissue | Mucosa of the colon |  |
| H17 | D15A1819-B30-C1 | Colorectal cancer | Adenocarcinoma | Ⅱ |
| H18 | D15A1819-B30-P1 | Adjacent tissue | Mucosa of the colon |  |
| I01 | D15A1992-B30-C1 | Colorectal cancer | Adenocarcinoma | Ⅱ |
| I02 | D15A1992-B30-P1 | Adjacent tissue | Mucosa of the colon |  |
| I03 | D15A1993-B30-C1 | Colorectal cancer | Adenocarcinoma | Ⅲ |
| I04 | D15A1993-B30-P1 | Adjacent tissue | Mucosa of the colon |  |
| I05 | D15A1820-B30-C1 | Colorectal cancer | Adenocarcinoma | Ⅱ |
| I06 | D15A1820-B30-P1 | Adjacent tissue | Mucosa of the colon |  |
| I07 | D15A1836-B30-C1 | Colorectal cancer | Adenocarcinoma | Ⅱ |
| I08 | D15A1836-B30-P1 | Adjacent tissue | Mucosa of the colon |  |
| I09 | D15A1839-B30-C1 | Colorectal cancer | Adenocarcinoma | Ⅱ |
| I10 | D15A1839-B30-P1 | Adjacent tissue | Mucosa of the colon |  |
| I11 | D15A1841-B30-C1 | Colorectal cancer | Adenocarcinoma | Ⅱ |
| I12 | D15A1841-B30-P1 | Adjacent tissue | Mucosa of the colon |  |
| I13 | D15A1904-B30-C1 | Colorectal cancer | Adenocarcinoma | Ⅲ |
| I14 | D15A1904-B30-P1 | Adjacent tissue | Mucosa of the colon |  |
| I15 | D15A1907-B30-C1 | Colorectal cancer | Adenocarcinoma | Ⅱ |
| I16 | D15A1907-B30-P1 | Adjacent tissue | Mucosa of the colon |  |
| I17 | D15A1914-B30-C1 | Colorectal cancer | Adenocarcinoma | Ⅲ |
| I18 | D15A1914-B30-P1 | Adjacent tissue | Mucosa of the colon |  |
| J01 | D15A1915-B30-C1 | Colorectal cancer | Adenocarcinoma | Ⅲ |
| J02 | D15A1915-B30-P1 | Adjacent tissue | Mucosa of the colon |  |
| J03 | D15A1917-B30-C1 | Colorectal cancer | Adenocarcinoma | Ⅰ |
| J04 | D15A1917-B30-P1 | Adjacent tissue | Mucosa of the colon |  |
| J05 | D15A1918-B30-C1 | Colorectal cancer | Adenocarcinoma | Ⅲ |
| J06 | D15A1918-B30-P1 | Adjacent tissue | Mucosa of the colon |  |
| J07 | D15A1919-B30-C1 | Colorectal cancer | Adenocarcinoma | Ⅲ |
| J08 | D15A1919-B30-P1 | Adjacent tissue | Mucosa of the colon |  |
| J09 | D15A1921-B30-C1 | Colorectal cancer | Adenocarcinoma | Ⅲ |
| J10 | D15A1921-B30-P1 | Adjacent tissue | Mucosa of the colon |  |
| J11 | D15A1923-B30-C1 | Colorectal cancer | Adenocarcinoma | Ⅲ |
| J12 | D15A1923-B30-P1 | Adjacent tissue | Mucosa of the colon |  |
| J13 | D15A1928-B30-C1 | Colorectal cancer | Adenocarcinoma | Ⅰ |
| J14 | D15A1928-B30-P1 | Adjacent tissue | Mucosa of the colon |  |
| J15 | D15A1929-B30-C1 | Colorectal cancer | Adenocarcinoma | Ⅲ |
| J16 | D15A1929-B30-P1 | Adjacent tissue | Mucosa of the colon |  |
| J17 | D15A1927-B30-C1 | Colorectal cancer | Adenocarcinoma | Ⅲ |
| J18 | D15A1927-B30-P1 | Adjacent tissue | Mucosa of the colon |  |

**CRC cohort 2**

| **Label** | **Histological type** | **Pathological type** | **Grade** |
| --- | --- | --- | --- |
| D15A0070 | Primary colon cancer | Adenocarcinoma | Ⅳ |
| D15A0311 | Primary colon cancer | Adenocarcinoma | Ⅳ |
| D15A3492 | Primary colon cancer | Papillary adenocarcinoma | Ⅳ |
| D15A3472 | Primary colon cancer | Adenocarcinoma | Ⅳ |
| D15A0447 | Primary colon cancer | Adenocarcinoma | Ⅳ |
| D15A0643 | Primary colon cancer | Adenocarcinoma | Ⅳ |
| D15A1070 | Primary colon cancer | Adenocarcinoma | Ⅳ |
| D15A1724 | Primary colon cancer | Adenocarcinoma | Ⅳ |
| D16A4335 | Primary rectal cancer | Adenocarcinoma | Ⅳ |
| D16A0763 | Primary rectal cancer | Adenocarcinoma | Ⅳ |
| D16A0876 | Primary rectal cancer | Adenocarcinoma | Ⅳ |
| D16A1332 | Primary rectal cancer | Adenocarcinoma | Ⅳ |
| D16A0417 | Primary rectal cancer | Adenocarcinoma | Ⅳ |
| D16A0492 | Primary rectal cancer | Adenocarcinoma | Ⅳ |
| D16A4836 | Primary rectal cancer | Adenocarcinoma | Ⅳ |
| D16A3532 | Primary rectal cancer | Adenocarcinoma | Ⅳ |
| D16A0105 | Primary rectal cancer | Adenocarcinoma | Ⅳ |
| D16A3519 | Primary rectal cancer | Adenocarcinoma | Ⅳ |
| D19A0284 | Metastatic colon cancer in liver | Metastatic Adenocarcinoma | Ⅳ |
| D19A2001 | Metastatic colon cancer in liver | Metastatic Adenocarcinoma | Ⅳ |
| D19A0699 | Metastatic colon cancer in liver | Metastatic Adenocarcinoma | Ⅳ |
| D19A0467 | Metastatic colon cancer in liver | Metastatic Adenocarcinoma | Ⅳ |
| D19A0411 | Metastatic colon cancer in liver | Metastatic Adenocarcinoma | Ⅳ |
| D19A0245 | Metastatic colon cancer in liver | Metastatic Adenocarcinoma | Ⅳ |
| D19A0079 | Metastatic colon cancer in liver | Metastatic Adenocarcinoma | Ⅳ |
| D19A0608 | Metastatic rectal cancer in liver | Metastatic Adenocarcinoma | Ⅳ |
| D19A0703 | Metastatic rectal cancer in liver | Metastatic Adenocarcinoma | Ⅳ |
| D19A0483 | Metastatic rectal cancer in liver | Metastatic Adenocarcinoma | Ⅳ |
| D19A2521 | Metastatic rectal cancer in liver | Metastatic Adenocarcinoma | Ⅳ |

**CRC cohort 3**

| **Label** | **Histological type** | **Pathological type** | **Grade** |
| --- | --- | --- | --- |
| 416 | Rectal cancer | Adenocarcinoma | Ⅲ |
| 417 | Left-sided colon cancer | Mucinous adenocarcinoma | Ⅱ |
| 426 | Rectal cancer | Adenocarcinoma | Ⅱ |
| 427 | Rectal cancer | Adenocarcinoma with mucinous adenocarcinoma | Ⅱ |
| 432 | Rectal cancer | Adenocarcinoma | Ⅱ |
| 433 | Rectal cancer | Adenocarcinoma | Ⅲ |
| 435 | Rectal cancer | Adenocarcinoma | Ⅲ |
| 436 | Right-sided colon cancer | Adenocarcinoma | NA |
| 437 | Right-sided colon cancer | Adenocarcinoma | Ⅱ |
| 438 | Rectal cancer | Adenocarcinoma | Ⅱ |
| 440 | Right-sided colon cancer | Adenocarcinoma | Ⅱ |
| 442 | Rectal cancer | Adenocarcinoma | Ⅲ |
| 447 | Transverse colon cancer | Adenocarcinoma | Ⅱ |
| 448 | Right-sided colon cancer | Adenocarcinoma with mucinous adenocarcinoma | Ⅱ |
| 452 | Rectal cancer | Adenocarcinoma with mucinous adenocarcinoma | Ⅲ |
| 456 | Rectal cancer | Adenocarcinoma | NA |
| 459 | Rectal cancer | Adenocarcinoma | Ⅲ |
| 460 | Left-sided colon cancer | Mucinous adenocarcinoma | Ⅲ |
| 464 | Rectal cancer | Adenocarcinoma | Ⅰ |
| 467 | Rectal cancer | Adenocarcinoma | Ⅰ |
| 468 | Sigmoid colon cancer | Adenocarcinoma | Ⅱ |
| 474 | Sigmoid colon cancer | Adenocarcinoma | Ⅲ |
| 475 | Rectal cancer | Adenocarcinoma with mucinous adenocarcinoma | Ⅲ |
| 485 | Colorectal cancer | Adenocarcinoma | Ⅲ |
| 507 | Sigmoid colon cancer | Adenocarcinoma | NA |
| 509 | Left-sided colon cancer | Adenocarcinoma | Ⅱ |
| 510 | Rectal cancer | Adenocarcinoma | Ⅲ |
| 515 | Sigmoid colon cancer | Adenocarcinoma | Ⅲ |
| 517 | Rectal cancer | Adenocarcinoma | Ⅲ |
| 522 | Right-sided colon cancer | Adenocarcinoma | Ⅱ |
| 536 | Rectal cancer | Adenocarcinoma | Ⅱ |
| 537 | Sigmoid colon cancer | Adenocarcinoma | Ⅱ |
| 538 | Transverse colon cancer | Adenocarcinoma | Ⅱ |
| 543 | Right-sided colon cancer | Adenocarcinoma | Ⅲ |
| 546 | Right-sided colon cancer | Adenocarcinoma | Ⅱ |
| 549 | Colorectal cancer | Adenocarcinoma with mucinous adenocarcinoma | NA |
| 556 | Right-sided colon cancer | Mucinous adenocarcinoma with signet-ring cell carcinoma | Ⅰ |
| 590 | Right-sided colon cancer | Adenocarcinoma | Ⅰ |
| 614 | Rectal cancer | Adenocarcinoma | Ⅰ |

**Supplementary Table 2 shRNA, siRNA sequence used in this study.**

**shRNA**

| **Target Gene** | **Sequences (5’-3’****)** |
| --- | --- |
| shSLC46A1#1 | CAGGAAACATTTAGCCCTCTA |
| shSLC46A1#2 | CCAGAGAAGTCCAGGAAACAT |

**siRNA**

| **Target Gene** | **Sequences (5’-3’)** | |
| --- | --- | --- |
| siSLC46A1#1 | sense | GAUCCAUUGUCCAGCUCUATT |
|  | antisense | UAGAGCUGGACAAUGGAUCTT |
| siSLC46A1#2 | sense | CUGAUUGGGAUGCUGGAAATT |
|  | antisense | UUUCCAGCAUCCCAAUCAGTT |
| siFOS#1 | sense | GGGUUCAUUAUUGGAAUUATT |
|  | antisense | UAAUUCCAAUAAUGAACCCTT |
| siFOS#2 | sense | GAGGUGGUCUGAAUGUUCUTT |
|  | antisense | AGAACAUUCAGACCACCUCTT |
| siPLAU#1 | sense | GCAUGACUUUGACUGGAAUTT |
|  | antisense | AUUCCAGUCAAAGUCAUGCTT |
| siPLAU#2 | sense | GGGAAACAUAAUUACUGCATT |
|  | antisense | UGCAGUAAUUAUGUUUCCCTT |

**Supplementary Table 3 Primers used in this study.**

| **Target Gene** | **Forward Primer (5'→3')** | **Reverse Primer (5'→3')** |
| --- | --- | --- |
| SLC46A1 | GCCCAGGACATCTTAACCCTTTA | AGCAACCCATATCCTGTGAACAT |
| FOS | AAGCGGAGACAGACCAACTAGAA | GATCAAGGGAAGCCACAGACATC |
| PLAU | GAGGGCAGCACTGTGAAATAGAT | TTGTCTGGGTTCCTGCAGTAATT |
| BCL2 | GGTGGGGTCATGTGTGTGG | CGGTTCAGGTACTCAGTCATCC |
| CCND1 | TGGAGCCCGTGAAAAAGAGC | TCTCCTTCATCTTAGAGGCCAC |
| GAPDH | TGTCAAGCTCATTTCCTGGTATG | TCTCTCTTCCTCTTGTGCTCTTG |

**Supplementary Table 4 Antibodies used in this study.**

| **Antibody** | **Company** | **Catalog No.** | **Dilution** |
| --- | --- | --- | --- |
| SLC46A1 | Santa Cruz | sc393460 | WB (1:2000); IHC (1:100); mIHC (1:100) |
| FOS | Cell Signaling Technology | #2250 | WB (1:1000); mIHC (1:100) |
| PLAU | Proteintech | 17968-1-AP | WB (1:4000); mIHC (1:100) |
| Pan-CK | Cell Signaling Technology | #4545S | mIHC (1:200) |
| Ki-67 | Cell Signaling Technology | #9449 | IHC (1:800) |
| GAPDH | Proteintech | 10494-1-AP | WB (1:2000) |

WB, western blot; IHC, immunohistochemistry; mIHC, multiplex immunohistochemistry.
