# Supplementary figures and images for: SLC46A1 deficiency-mediated folate restriction suppresses colorectal cancer progression through epigenetic-transcriptional reprogramming
Source: Cell Death Dis. 2026 Jan 31;17(1):189. doi: 10.1038/s41419-026-08423-8 (PMC12876983; doi:10.1038/s41419-026-08423-8)

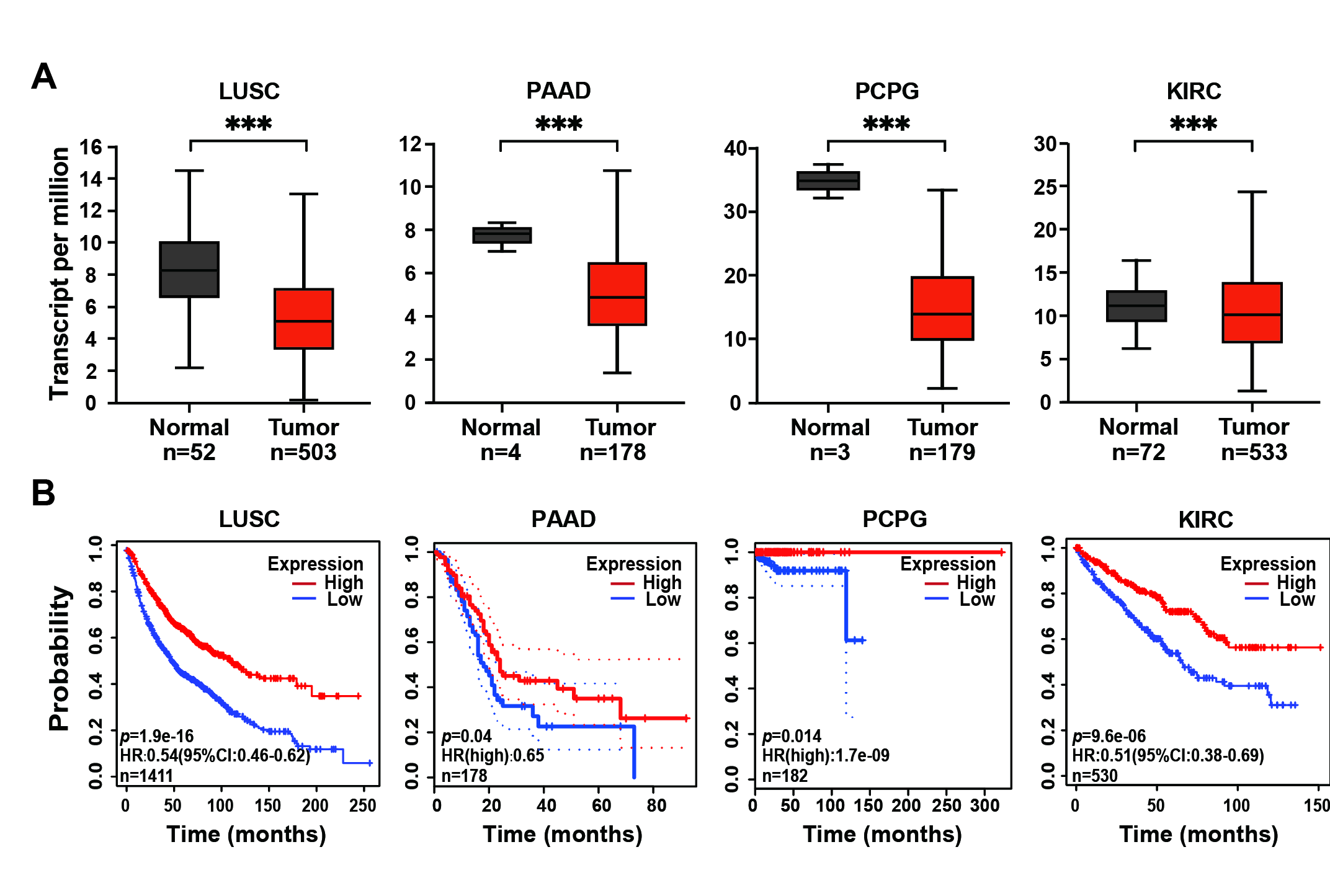

Supplement: Supplementary file 3 — Supplementary Figure 1 [file 41419_2026_8423_MOESM3_ESM.tif]

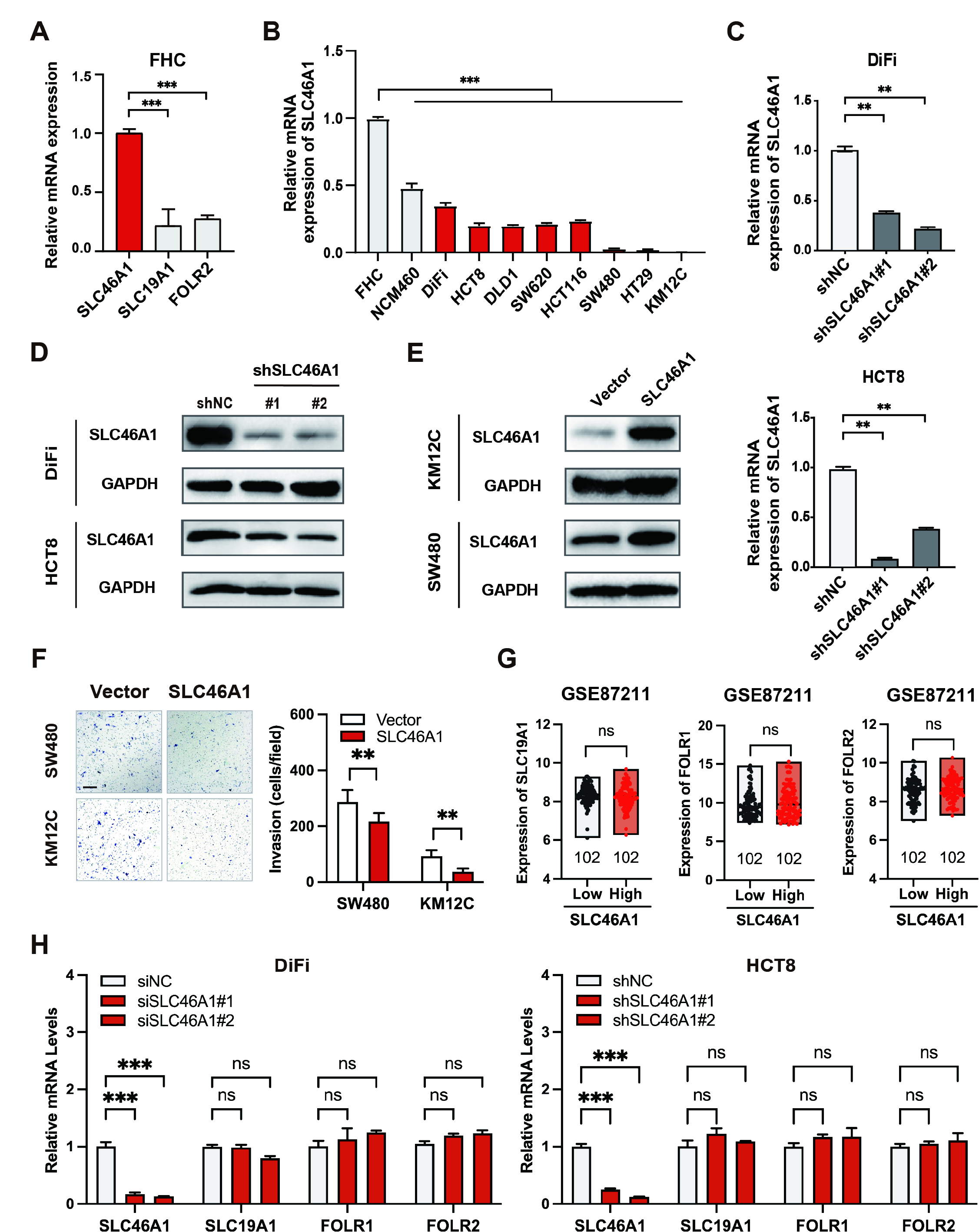

Supplement: Supplementary file 4 — Supplementary Figure 2 [file 41419_2026_8423_MOESM4_ESM.tif]

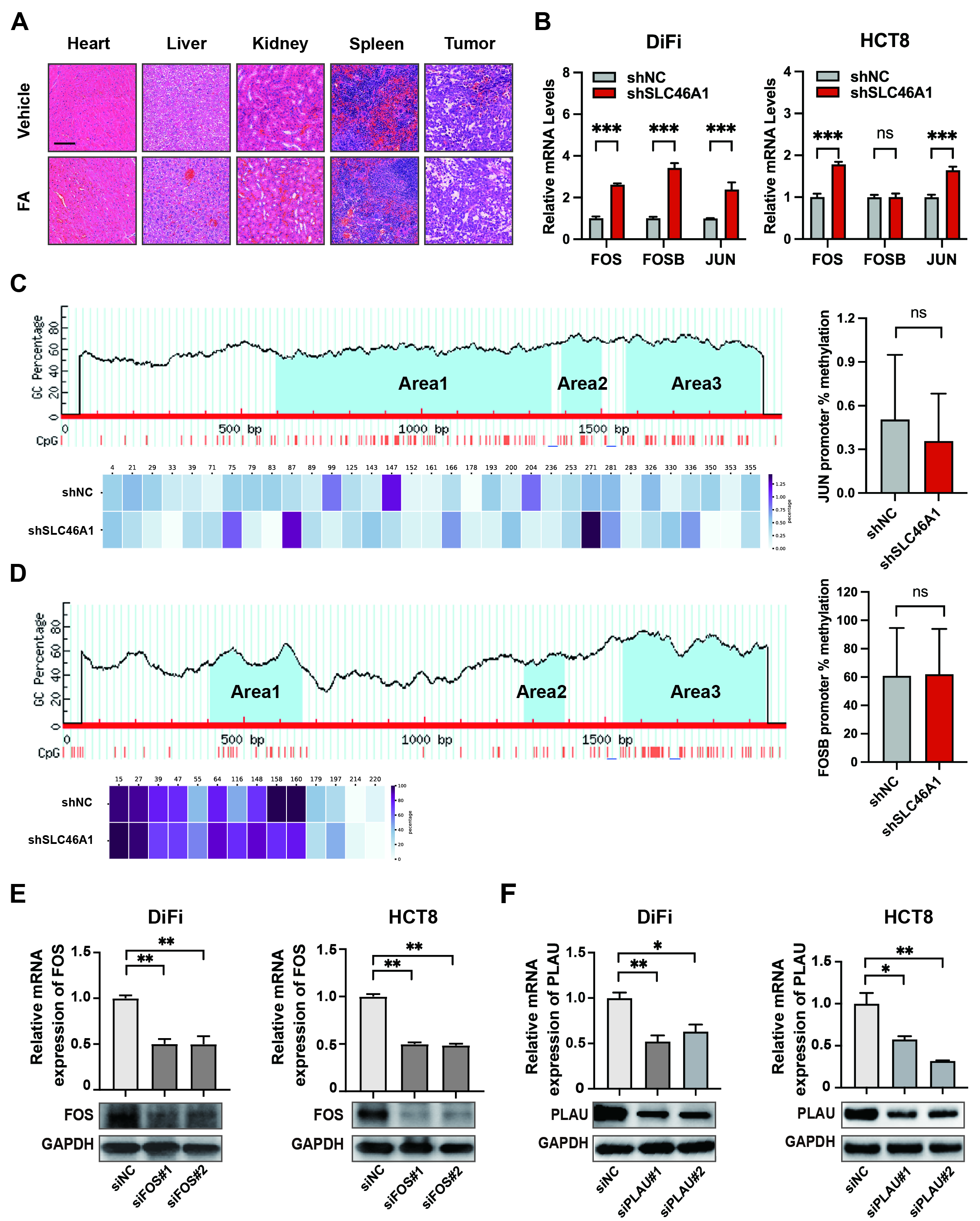

Supplement: Supplementary file 5 — Supplementary Figure 3 [file 41419_2026_8423_MOESM5_ESM.tif]
