# Supplementary material for: SLC46A1 deficiency-mediated folate restriction suppresses colorectal cancer progression through epigenetic-transcriptional reprogramming
Source: Cell Death Dis. 2026 Jan 31;17(1):189. doi: 10.1038/s41419-026-08423-8 (PMC12876983; doi:10.1038/s41419-026-08423-8)
Supplement: Supplementary file 6 — Full and uncropped western blots [file 41419_2026_8423_MOESM6_ESM.docx]

**Figure 4-D**

**DiFi**

SLC46A1 FOS JUN GAPDH


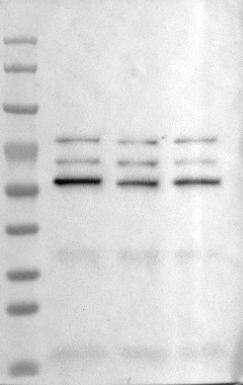

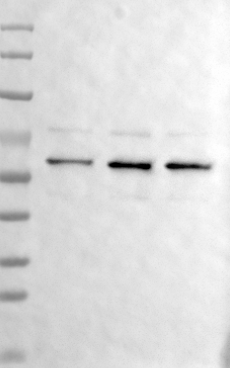

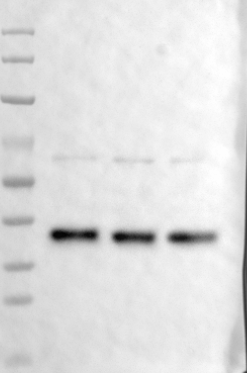

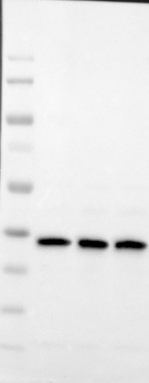


**HCT8**

SLC46A1 FOS JUN GAPDH

**
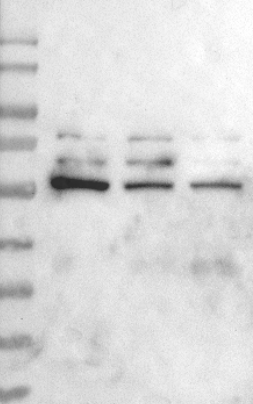

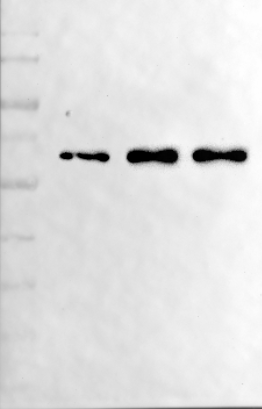

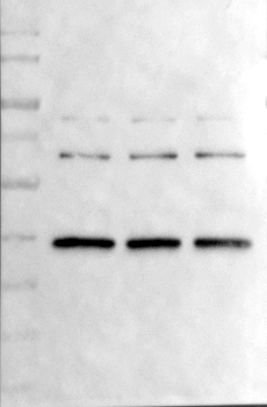

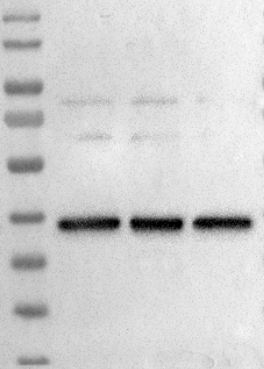
**

**Figure 4- F**

**DiFi**

SLC46A1 FOS GAPDH


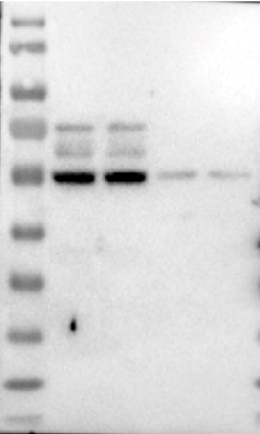

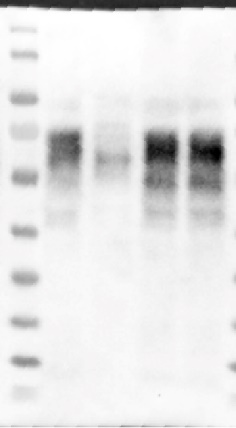

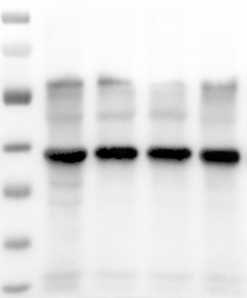


**HCT-8**

SLC46A1 FOS GAPDH


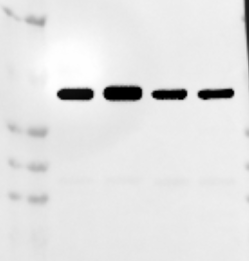

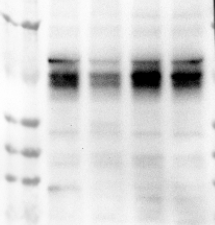

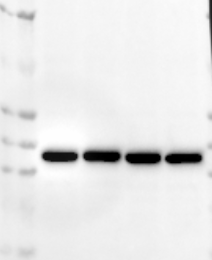


**Figure 6 – A**

**DiFi HCT-8**

PLAU GAPDH PLAU GAPDH


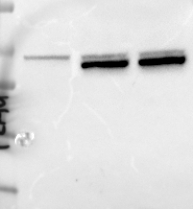

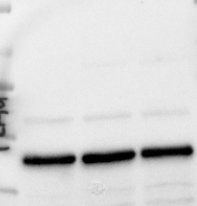

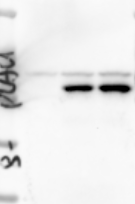

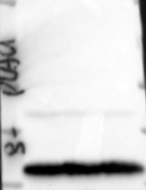


**Figure 6 – B**

**DiFi HCT-8**

PLAU GAPDH PLAU GAPDH


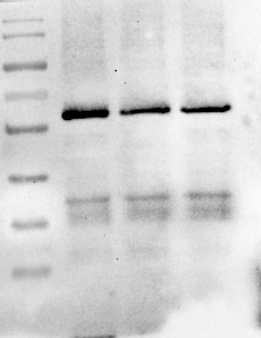

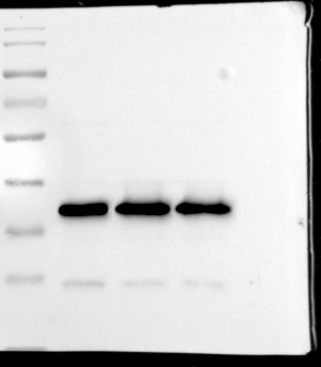

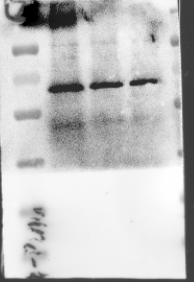

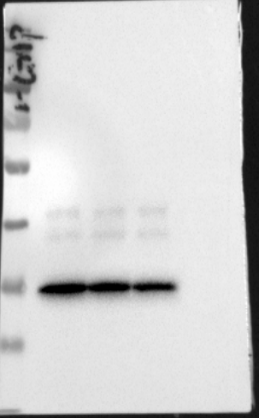


**Figure 6 – D**

**DiFi**

SLC46A1 FOS PLAU GAPDH


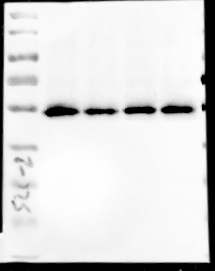

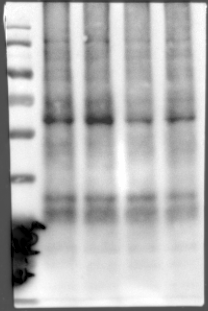

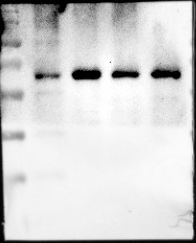

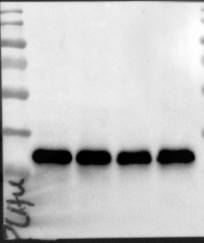


**HCT8**

SLC46A1 FOS PLAU GAPDH


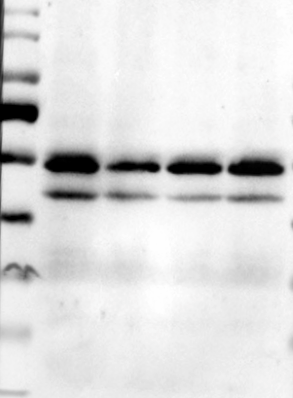

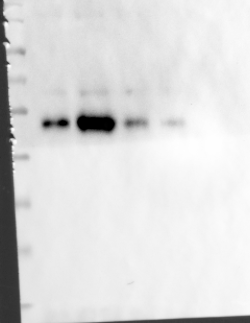

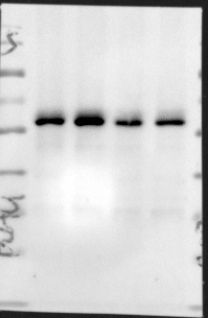

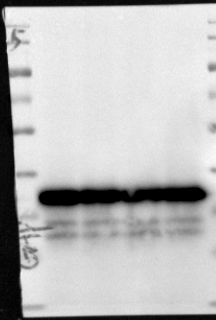


**Figure 6 – F**

**DIFI**

SLC46A1 PLAU GAPDH


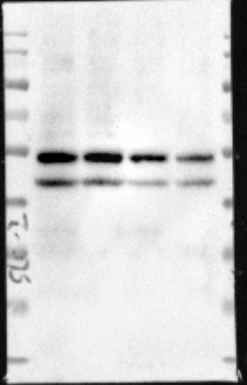

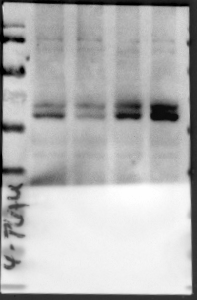

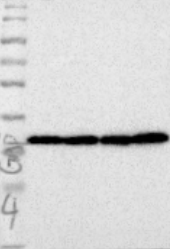


**HCT-8**

SLC46A1 PLAU GAPDH
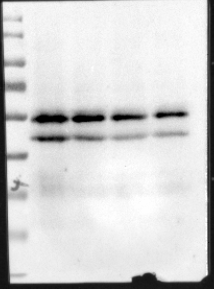

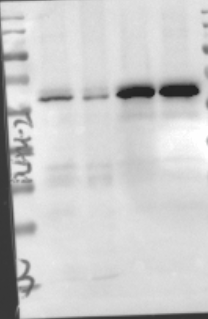

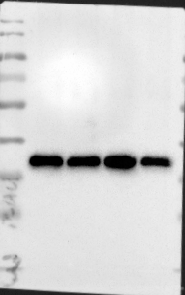


**Figure S2 – D**

**DiFi HCT8**

SLC46A1 GAPDH SLC46A1 GAPDH


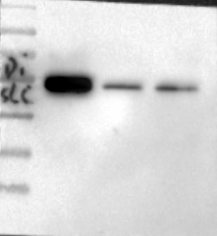

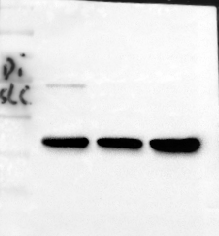

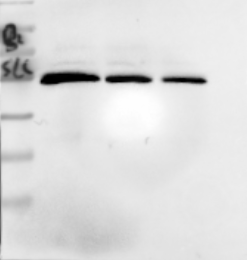

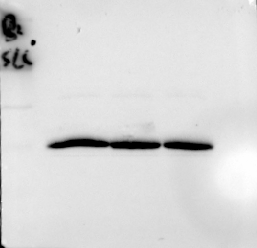


**Figure S2 - E**

**KM12C SW480**

SLC46A1 GAPDH SLC46A1 GAPDH

**
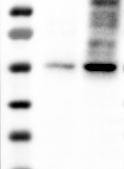
**
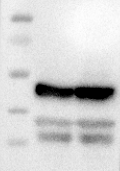
 **
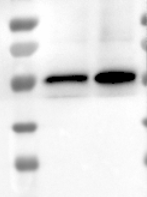
**
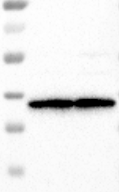


**Figure S3 – E**

**DiFi HCT-8**

FOS GAPDH FOS GAPDH


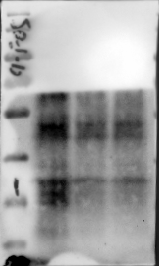
 **
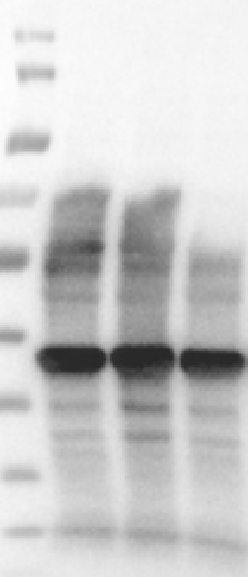

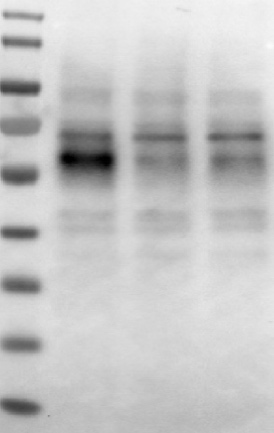

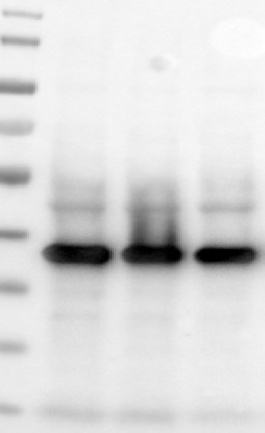
**

**Figure S3 – F**

**DiFi HCT-8**

PLAU GAPDH PLAU GAPDH


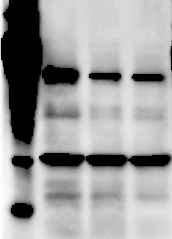

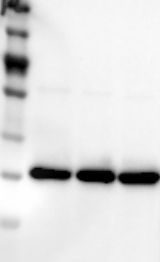
 **
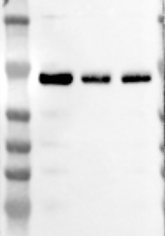

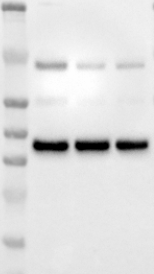
**
